# Supplementary material for: Thio-NHS esters are non-innocent protein acylating reagents
Source: Nat Commun. 2025 Jul 1;16:6028. doi: 10.1038/s41467-025-60527-5 (PMC12217259; doi:10.1038/s41467-025-60527-5)
Supplement: Supplementary file 1 — Supplementary Information [file 41467_2025_60527_MOESM1_ESM.pdf]

Supplementary Materials for

NHS esters are non-innocent protein acylating reagents

TABLE OF CONTENTS

Supplementary Figures 3

Supplementary Tables 45

Supplementary Experimental Procedures 47

Reagent and materials 47

General considerations 47

Chemical synthesis 48

Supplementary References 61

Supplementary Figures

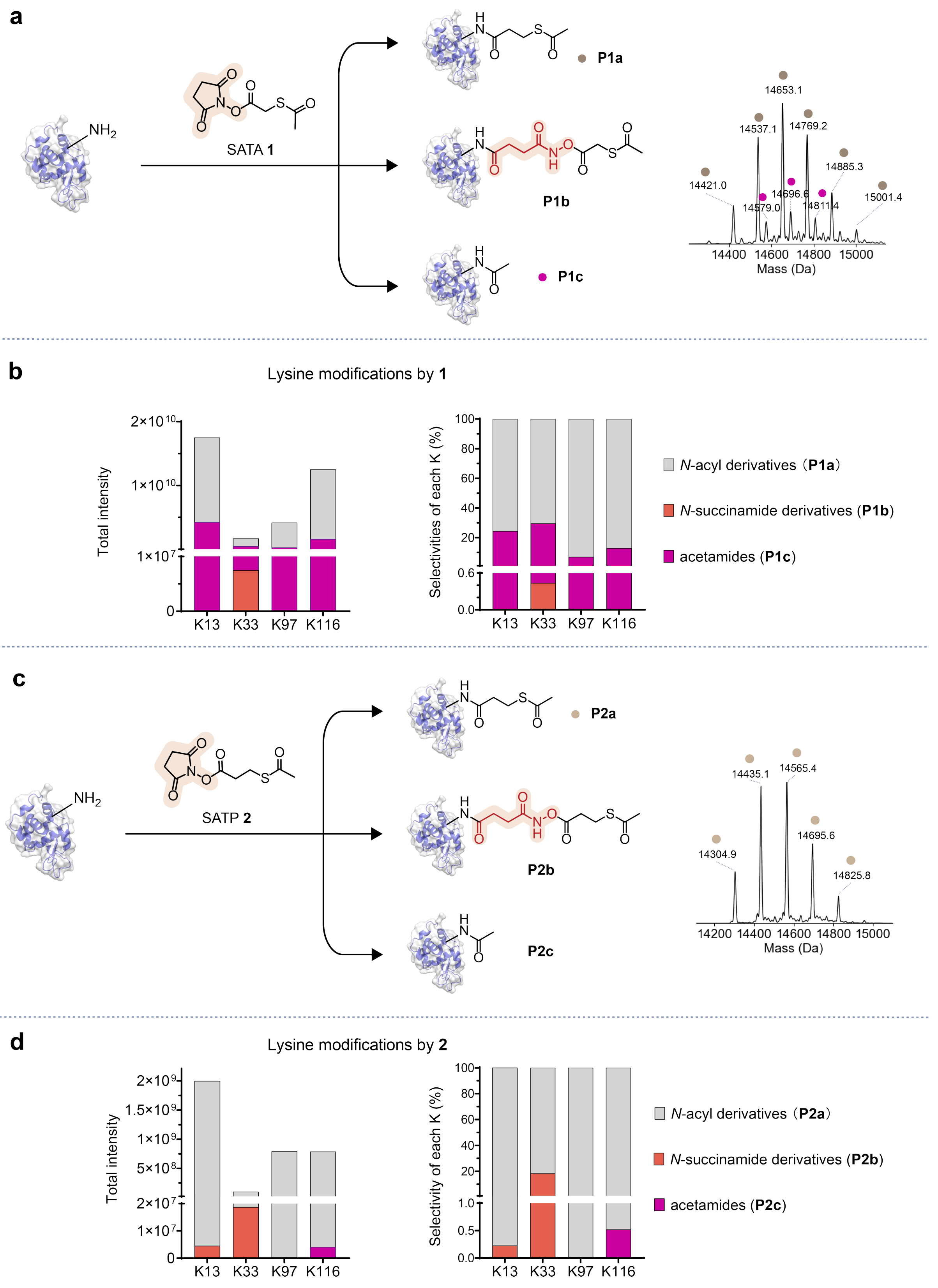

Supplementary Fig. 1 | Evaluation of the coupling reaction of SATA **1** and SATP **2** with the HEL protein by LC-MS and tandem mass spectrometry. The experiment was conducted for three independent biological replicates with representative data shown. (**a**) LC-MS analysis of reactions of HEL with SATA **1**. (**b**) The peptide abundances of different lysine modifications induced by SATA **1**. (**c**) LC-MS analysis of reactions of HEL with SATP **2**. (**d**) The peptide abundances of different lysine modifications induced by SATP **2**. Reagents and conditions: 60 equivalents of **1** or **2** (respect to Lys), 1 mg/mL HEL, 0.1 M $NaHCO_3$, r.t, 30 min. Source data are provided as a Source Data file.

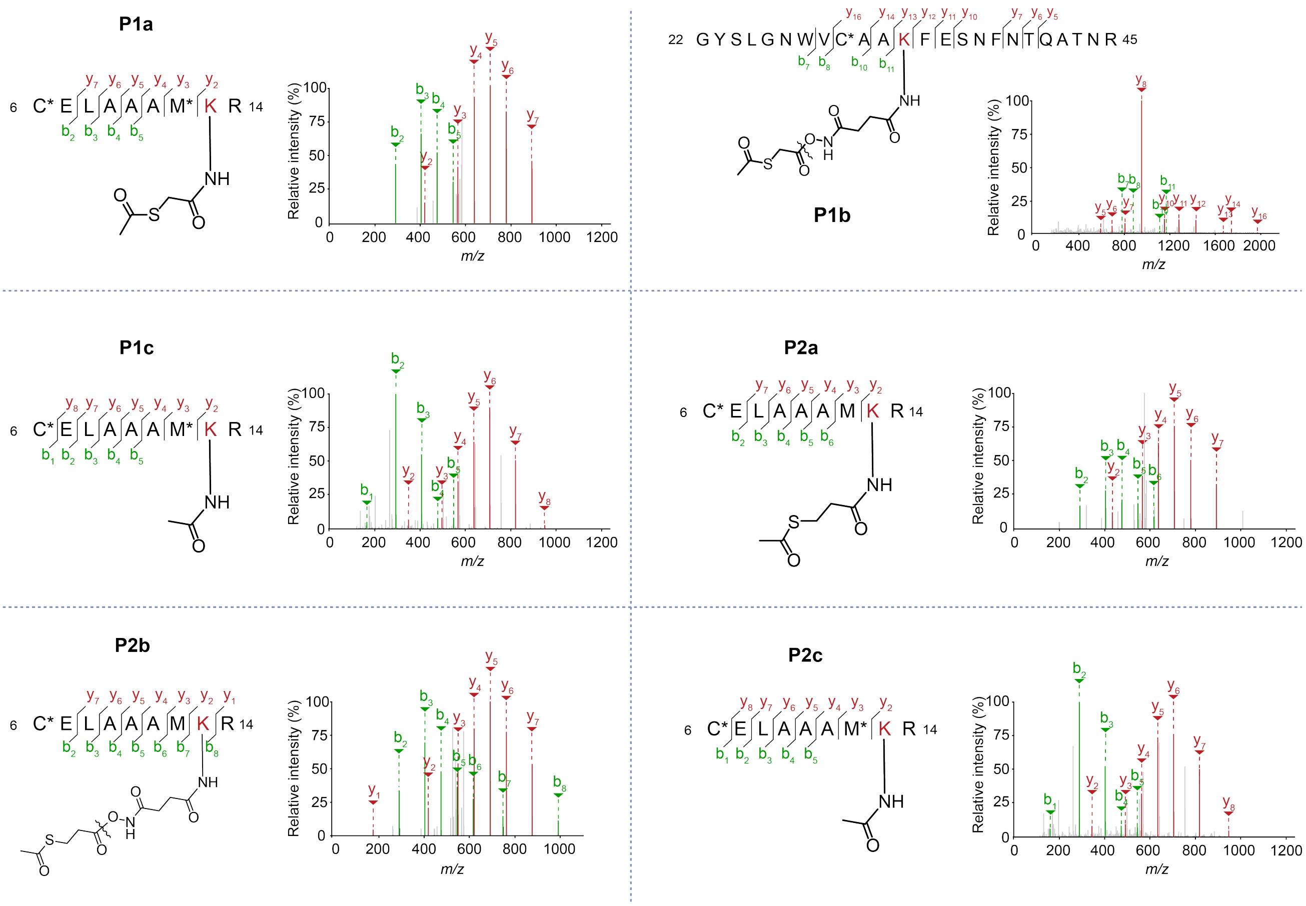

Supplementary Fig. 2 | Tandem mass spectrum of selected derivatized peptide precursors detected in full-scan mass spectra, including *N*-acyl derivatized peptide precursors (**P1a**, +115.9908 and **P2a**, +130.0048), *N*-succinamide derivatized peptide precursors (**P2a**, +115.0230), and acetylated derivatized peptide precursors (**P1c**, +42.0206； **P2c**, +42.0099). Representative results are shown from three independent biological replicates. C* denotes the carbamidomethylation of cysteine, M* denotes the oxidation of methionine. Source data are provided as a Source Data file.

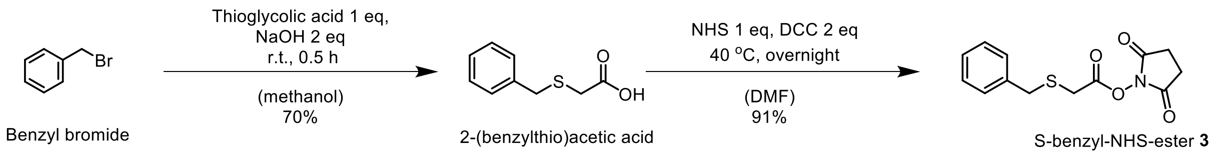

Supplementary Fig. 3 | Synthesis route of S-benzyl-NHS-ester **3**.

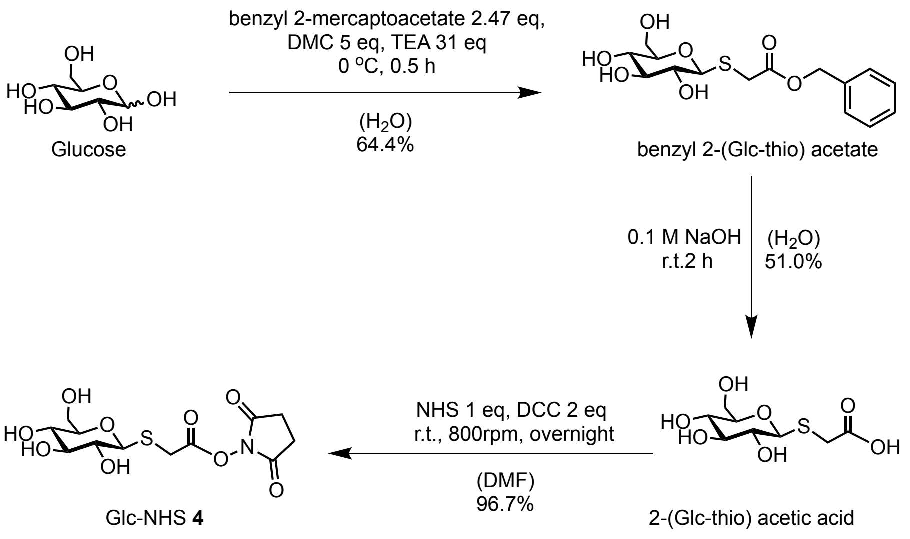

Supplementary Fig. 4 | Synthesis route of Glc-NHS **4**.

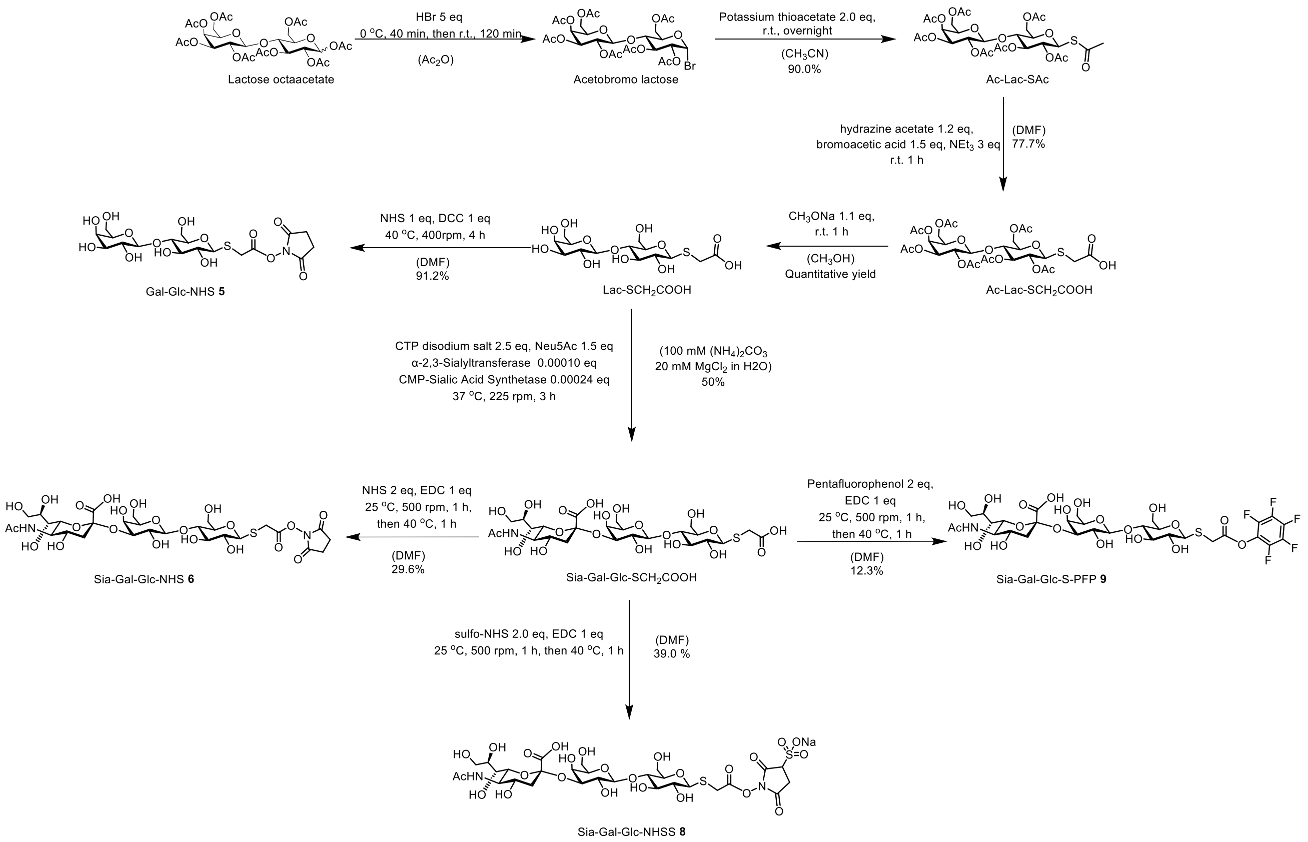

Supplementary Fig. 5 | Synthesis routes for Gal-Glc-NHS **5**, Sia-Gal-Glc-NHS **6**, Sia-Gal-Glc-NHSS **8**, and Sia-Gal-Glc-S-PFP **9**.

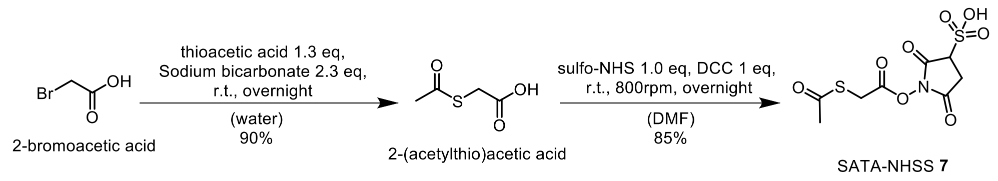

Supplementary Fig. 6 | Synthesis route for SATA-NHSS **7**.

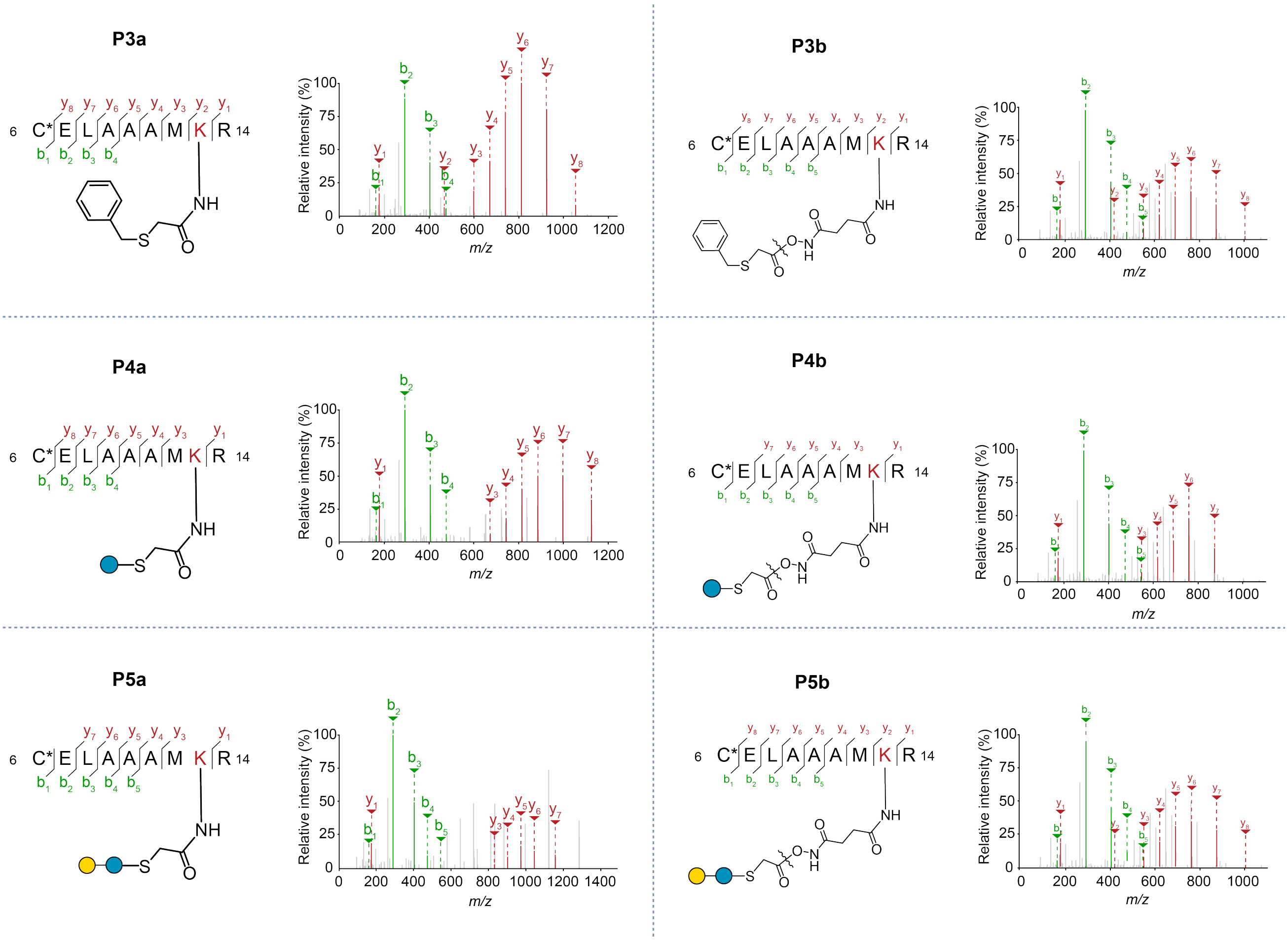

Supplementary Fig. 7 | Tandem mass spectra of selected derivatized peptide precursors detected in full-scan mass spectra, including *N*-acyl derivatized peptide precursors (**P3a,** +164.0294, **P4a**, +236.0351, and **P5a**, +398.0869) and *N*-succinamide derivatized peptide fragments (**P3b**, +115.0278; **P4b**, +115.0269; and **P5b,** +115.0274). Proteomic analyses were conducted twice for **5a** and once for **3a** and **4a** with similar results (several peptides in each dataset display the described behaviour). Representative spectra for the same peptide across experiments are shown. C* denotes the carbamidomethylation of Cysteine, M* denotes the oxidation of Methionine. Source data are provided as a Source Data file.

a

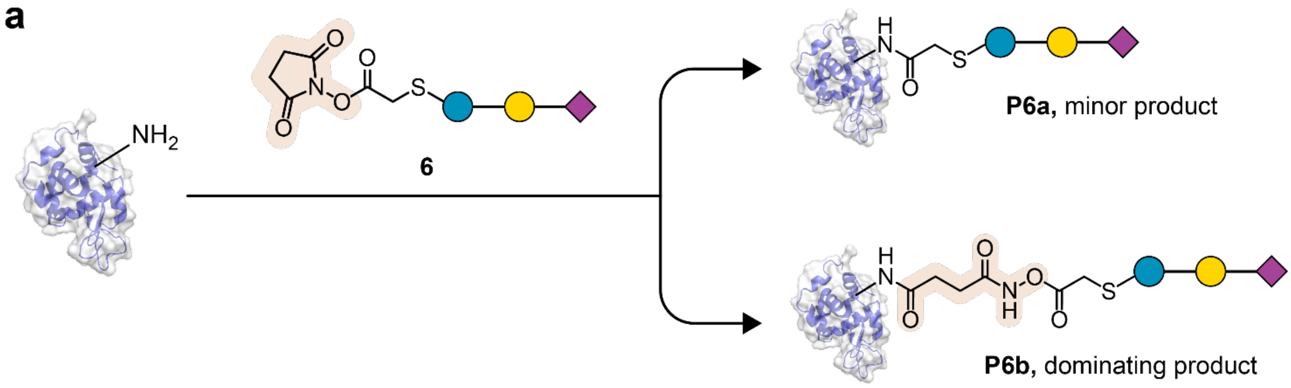

b

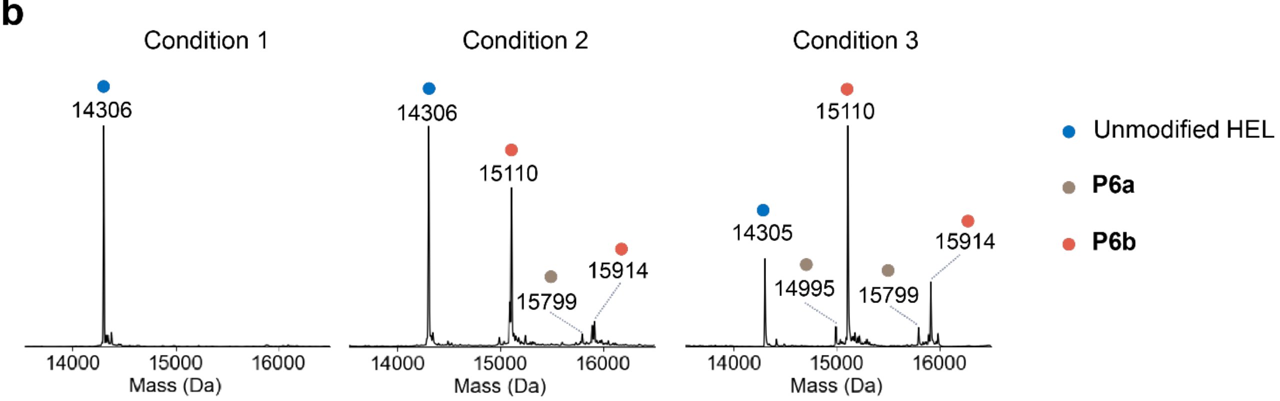

Conditions	Buffer	Equiv.[1]	Yield of P6a[2]	Yield of P6b[3]
1	PBS (pH = 7.23)	200	ND	ND
2	0.1 M $NaHCO_3$ in PBS (pH = 8.42)	200	4%	42%
3	0.1 M $NaHCO_3$ (pH = 8.51)	200	10%	71%

c

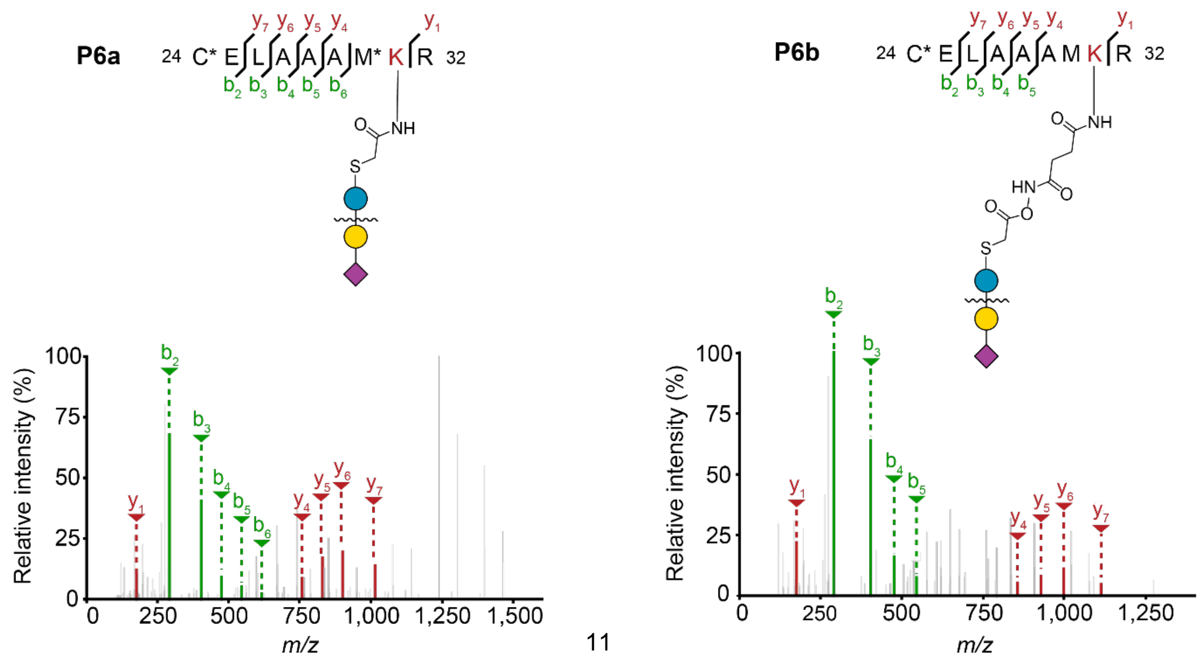

Supplementary Fig. 8 | Sia-Gal-Glc-NHS **6** predominantly yielded the *N*-succinamide derivative upon reaction with hen egg lysozyme (HEL) protein. (**a**) **6** affords *N*-succinamide derivative **P6b** (major product) as well as the *N*-acyl derivative **P6a** (minor product). (**b**) Mass spectra of the reaction mixtures obtained from LC-MS analysis under different reactions as show in the table. ND, not detected. [1]Equiv. with respect to lysine. [2,3]Include singly and doubly modified HEL. Reagents and conditions: 200 equivalents of **6** (respect to Lys), 1 mg/mL HEL, r.t, 2 h. Representative results are shown from three independent biological replicates. (**c**) Identification of lysine residue modification by **6** using tandem mass spectrometry. Left panel, tandem mass spectrum of a selected acylated peptide precursor ion (+689.1831, **P6a**). Right panel, tandem mass spectrum of a selected *N*-succinamide derivatized peptide precursor ion (+804.2099, **P6b**) detected in full-scan mass spectra. Neutral losses of α-Neu5Ac-(2→3)-β-Gal (+453.1374) from the MS1 precursors were observed to due to the HCD fragmentation. Representative spectra are shown from three replicates. C* denotes the carbamidomethylation of Cysteine, M* denotes the oxidation of Methionine. Source data are provided as a Source Data file.

a

Conditions	Buffer	Equiv. of 6 (relative to -NH_2)	Temp.	HEL Conc (mg/mL)
1	0.1 M $NaHCO_3$ in PBS	50	rt	1
2	0.1 M $NaHCO_3$ in PBS	100	rt	1
3	0.1 M $NaHCO_3$ in PBS	200	rt	1
4	0.1 M $NaHCO_3$	200	rt	1
5	0.1 M $NaHCO_3$	100	rt	2
6	0.1 M $NaHCO_3$	100	rt	5
7	0.1 M $NaHCO_3$	50	0 ℃	1
8	0.1 M $NaHCO_3$	50	37 ℃	1
9	0.1 M $NaHCO_3$+ 10% DMF	100	rt	2
10	0.1 M $NaHCO_3$+ 10% DMF	100	rt	5
11	0.1 M $NaHCO_3$ + 10% DMSO	100	rt	1

b

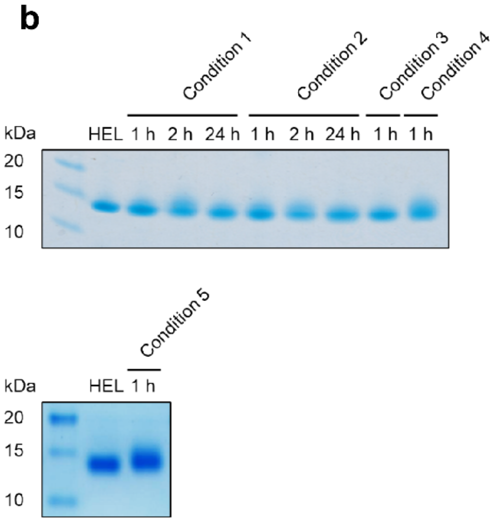

c

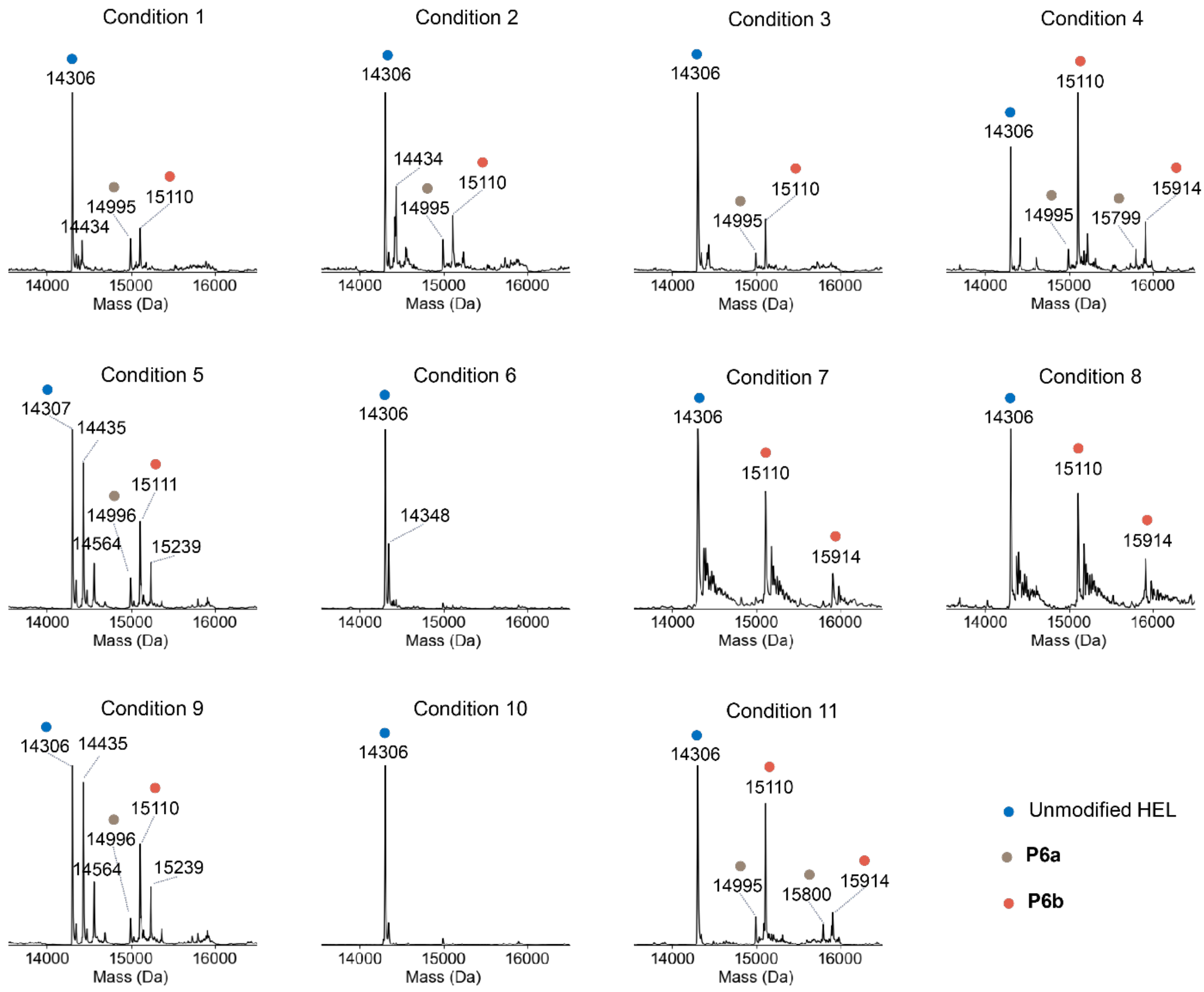

Supplementary Fig. 9 | Sia-Gal-Glc-NHS **6** resulted in the predominant formation of *N*-succinamide derivative under various conditions. (**a**) Coupling reaction conditions used to couple 1.0 mg/mL hen egg lysozyme (HEL) and **6**. Equivalents of **6** (respect to Lys) are

indicated. Reaction conducted for 2 h. (**b**) Verification of lysine modification in the HEL protein, as examined by SDS-PAGE. Representative results are shown from three replicates. (**c**) Mass spectra of the reaction mixtures obtained from LC-MS analysis under different reactions as show in (**a**). Source data are provided as a Source Data file.

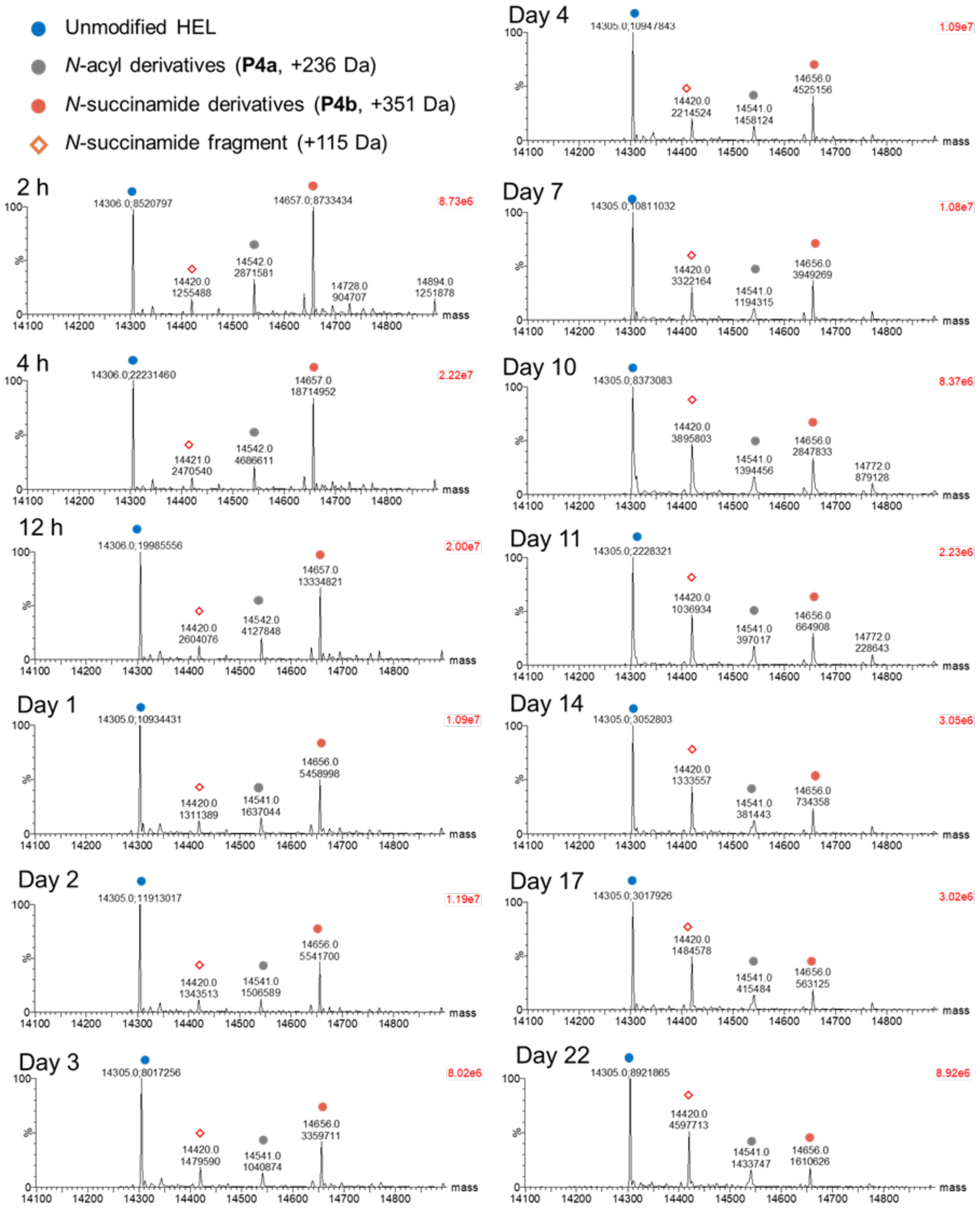

Supplementary Fig. 10 | Stability assessment of Glc-NHS 4 modified HEL products. HEL was reacted with 33 equivalents (33 eq respect to Lys) of Glc-NHS **4** at 0 °C for 2 h, followed by desalting. The protein was stored at 4 °C and monitored via LC-MS at specific time points. The first label on each peak represents the molecular weight, and the second represents intensity. For the stability curve fit (**Fig. 2g**), the conversion of each product is normalized by dividing the peak intensity by the sum of intensities for all peaks. Representative results are shown from three replicates. Source data are provided as a Source Data file.

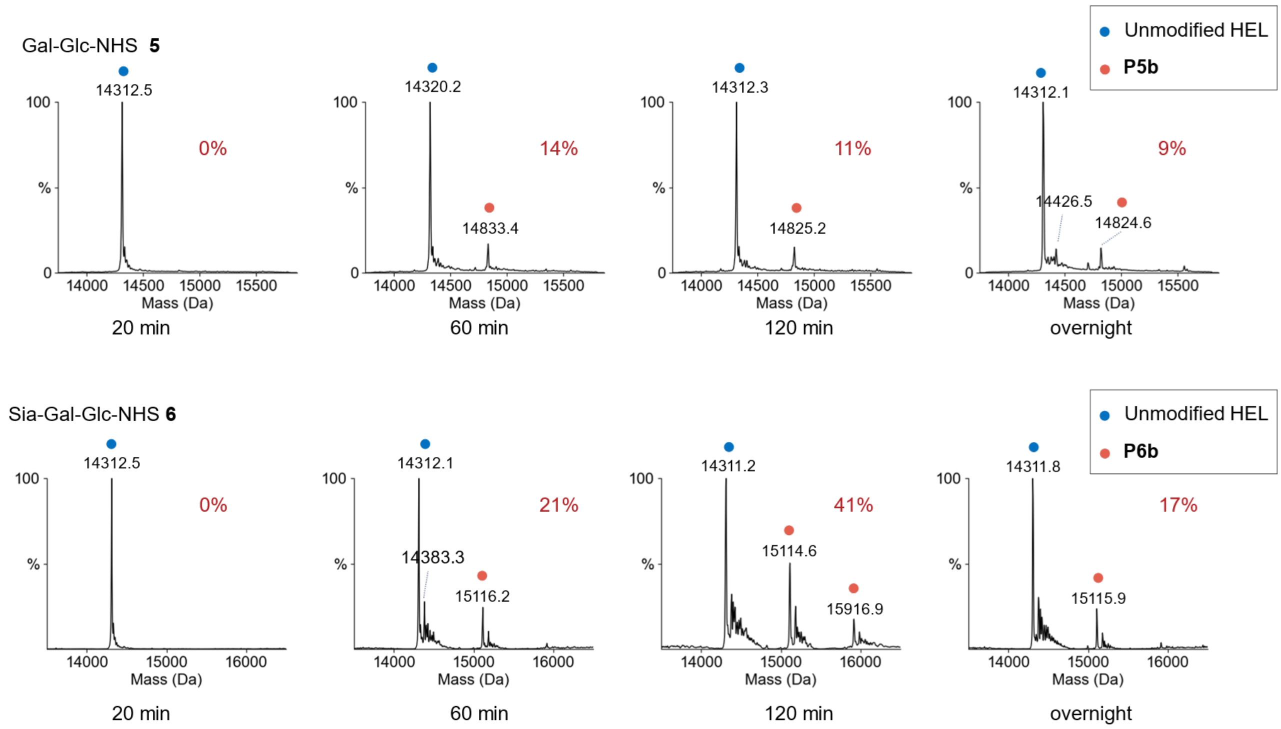

Supplementary Fig. 11 | Stability assessment of Gal-Glc-NHS 5 and Sia-Gal-Glc-NHS 6 modified HEL products. Reaction condition: 200 equivalents of **4-6** (respect to Lys), 1.0 mg/mL HEL, 0.1 M $NaHCO_3$ in PBS, 0 °C, reaction conducted for 20 min, 60 min, 120 min or overnight. Notably the level of modification initially increased and then decreased over time. Representative results are shown from three replicates. Source data are provided as a Source Data file.

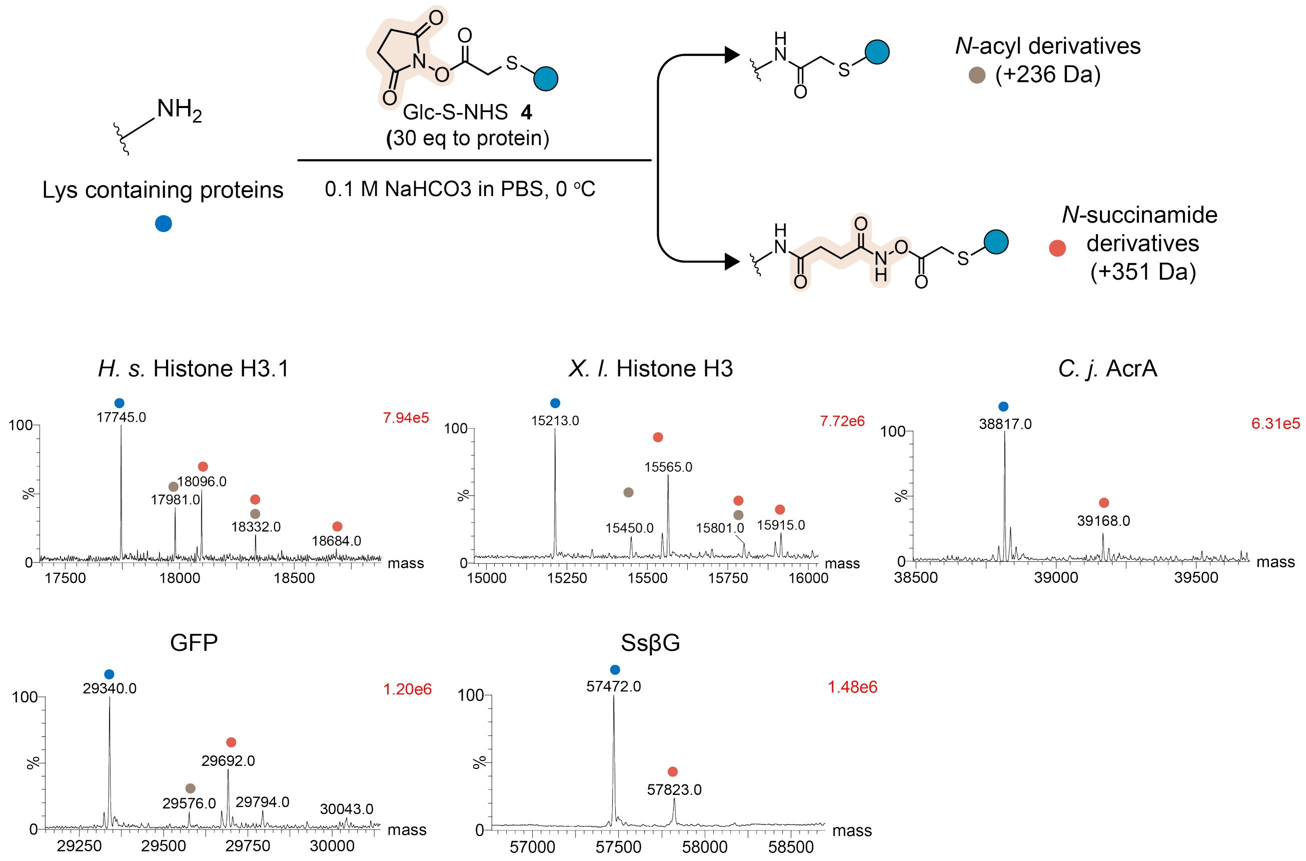

Supplementary Fig. 12 | Evaluation of the coupling reaction of Glc-NHS 4 with different protein substrates. Proteins with different lysine environment complexity confer *N*-acyl derivatives and *N*-succinamide derivatives. Conditions: 200 equivalents of **4** (respect to proteins), 0.1 M $NaHCO_3$ in PBS, 0 °C, 0.5 h. Representative results are shown from at least three replicates. Source data are provided as a Source Data file.

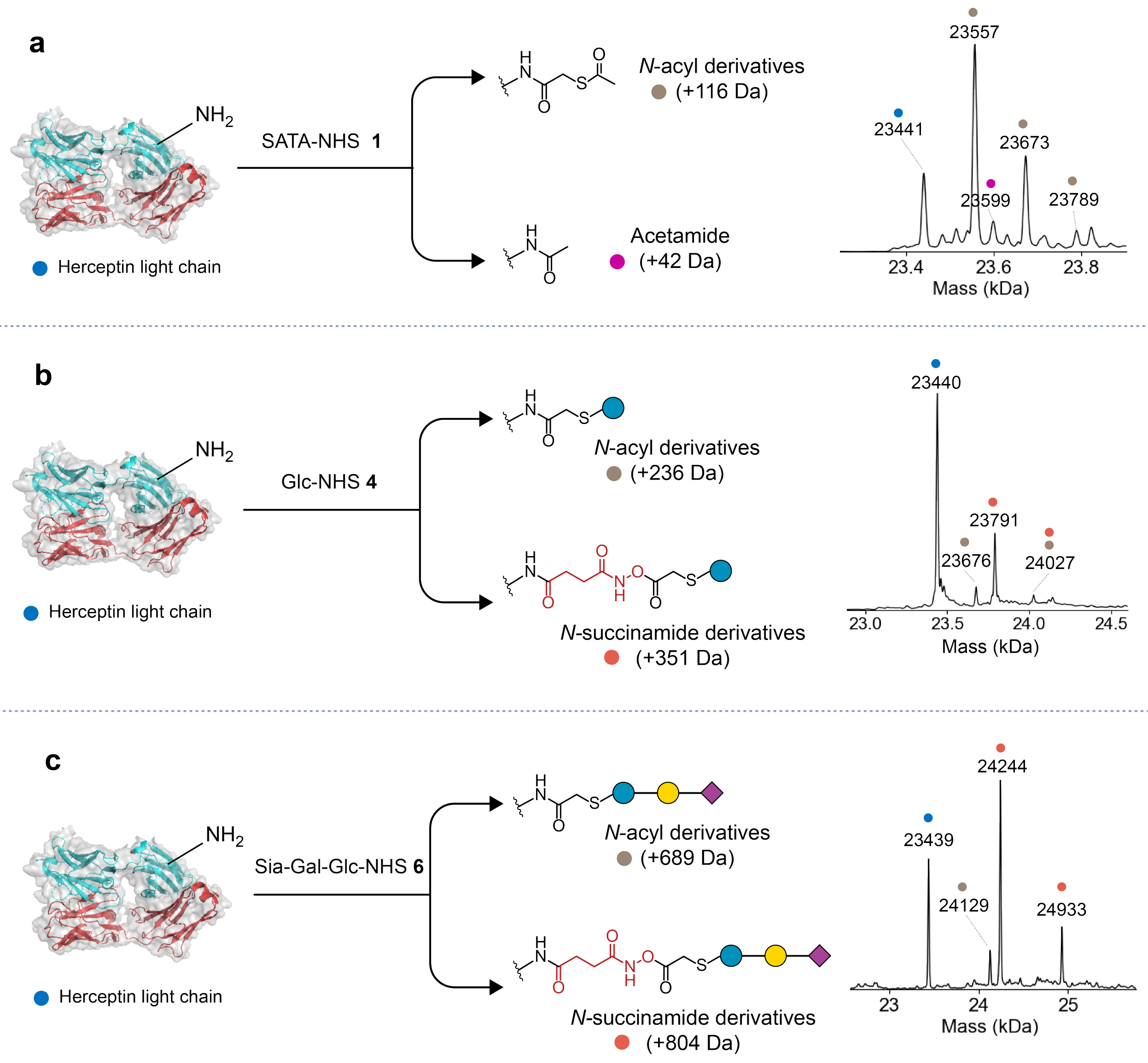

Supplementary Fig. 13 | Evaluation of the coupling reaction of SATA-NHS 1, Glc-NHS 4, and Sia-Gal-Glc-NHS 6 with Herceptin Fab. Conditions: 200 equivalents of **1**, **4**, or **6** (respect to proteins), 1.8 mg/mL Herceptin, 0.1 M $NaHCO_3$ in PBS, 0 °C, 0.5 h for **1** and **4**, 2 h for **6**. Representative results are shown from at least three replicates. Source data are provided as a Source Data file.

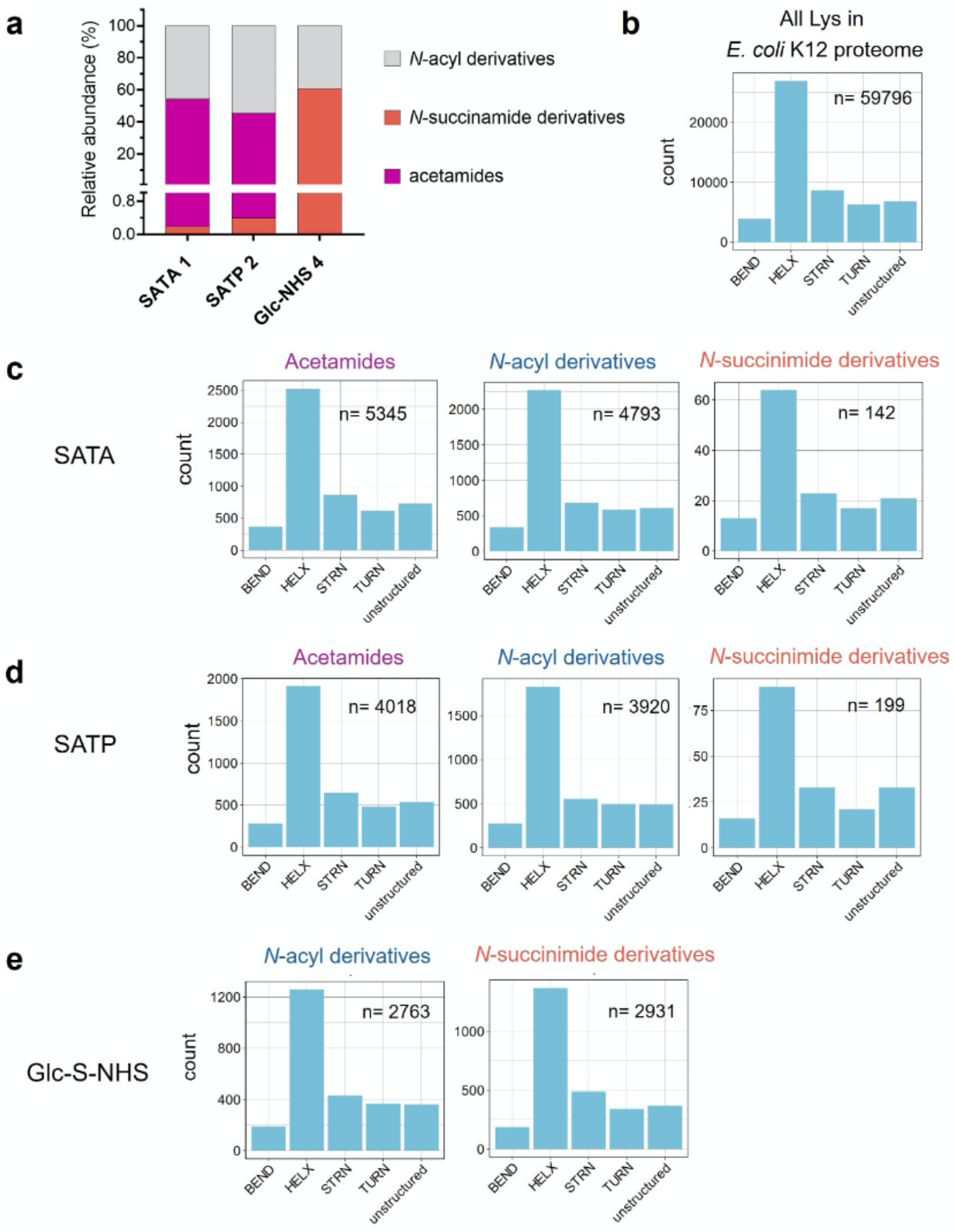

Supplementary Fig. 14 | Proteomic analysis of lysines that are modified by SATA 1, SATP 2, and Glc-NHS 4. (**a**) Abundances of the three lysine derivatives identified by tandem mass spectrometry, normalized by dividing each derivative's modified lysine count by the total modified lysine count (1, n=6826; 2, n=5598; 3, n=3752). (**b-e**) Distribution of lysines in *E. coli* (**b**) and their modifications by SATA (**c**), SATP (**d**), and Glc-NHS (**e**) across secondary structures: Bend, Helix (alpha-helix), STRN (strand), TURN, unstructured. Source data are provided as a Source Data file.

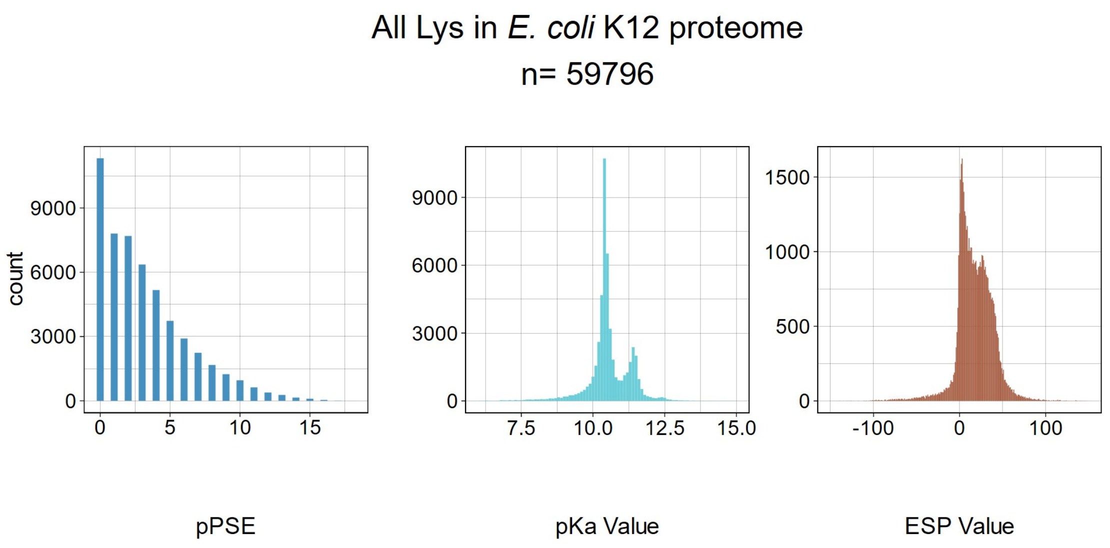

Supplementary Fig. 15 | Distributions of all 59796 lysines in *E. coli* proteome. AlphaFold-enabled pPSE (prediction-aware part-sphere exposure), pKa, and ESP (electrostatic potential) as measures of accessibility, charge and polarity, respectively. Low-confidence predictions (prediction quality ≤70) were removed. Source data are provided as a Source Data file.

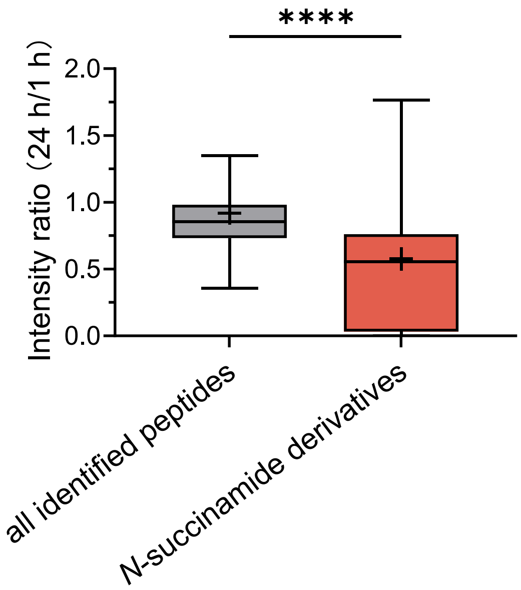

Supplementary Fig. 16 | Intensity ratio comparison of identified peptides and *N*-succinamide modified peptides after Glc-NHS 4 labeling at 1 h and 24 h. A significant decrease in intensity for *N*-succinamide modified peptides was shown after 24 h. ****p<0.0001 (Mann-Whitney test. Compare rank: unpaired, assume no Gaussian distribution, use nonparametric test). Source data are provided as a Source Data file.

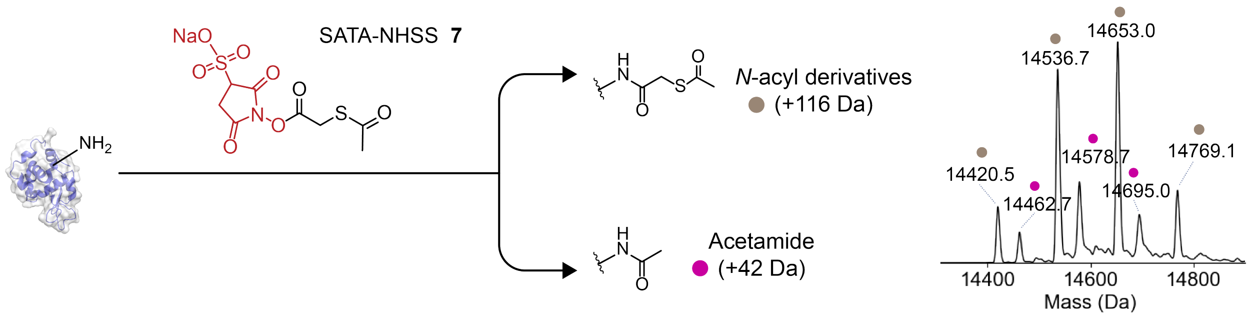

Supplementary Fig. 17 | LC-MS analysis of reactions of HEL with SATA-NHSS 7. Reagents and conditions of: 200 equivalents of **7** (respect to Lys), 1 mg/mL HEL, 0.1 M $NaHCO_3$ in PBS, 0 °C, 30 min. Representative results are shown from three replicates. Source data are provided as a Source Data file.

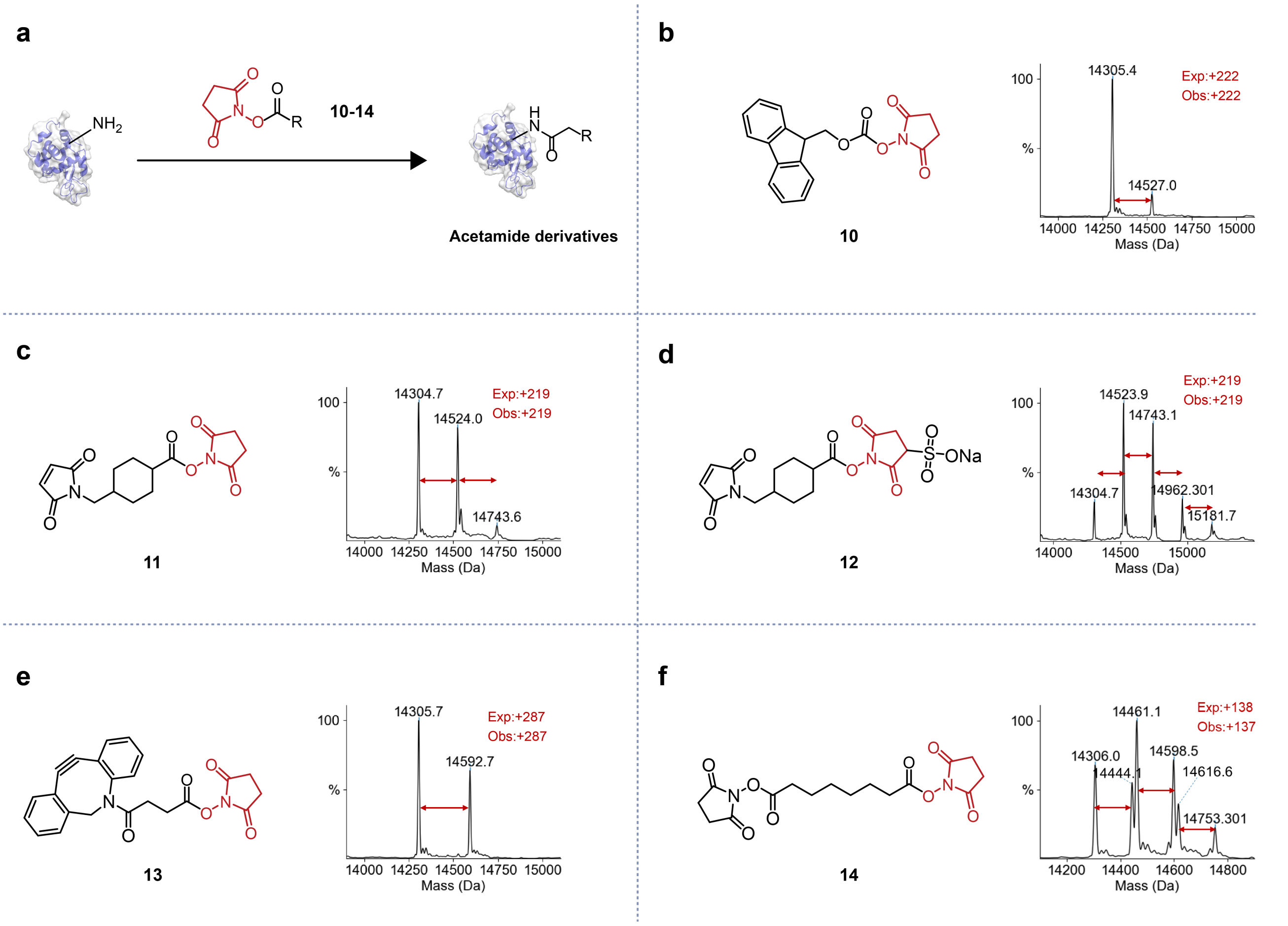

Supplementary Fig. 18 | Commercial NHS esters devoid of sulfonyl substituents gave rise to the *N*-acyl derivative exclusively upon their reaction with HEL protein. Reagents and conditions: 200 equivalents of commercial NHS esters (respect to Lys), 1 mg/mL HEL, 0.1 M $NaHCO_3$ in PBS, 0 °C, 1 h. Source data are provided as a Source Data file.

2-(benzylthio)acetic acid (^{1}H NMR 601 MHz, $CDCl_3$)

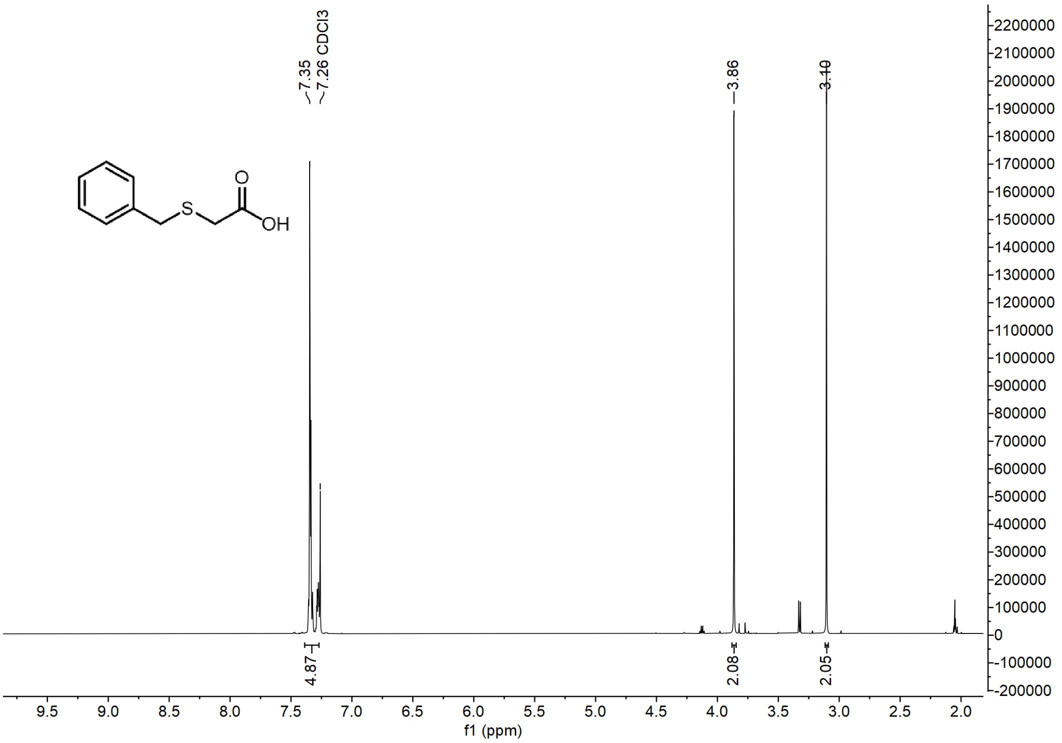

2-(benzylthio)acetic acid (^{13}C NMR 151 MHz, CDCl3)

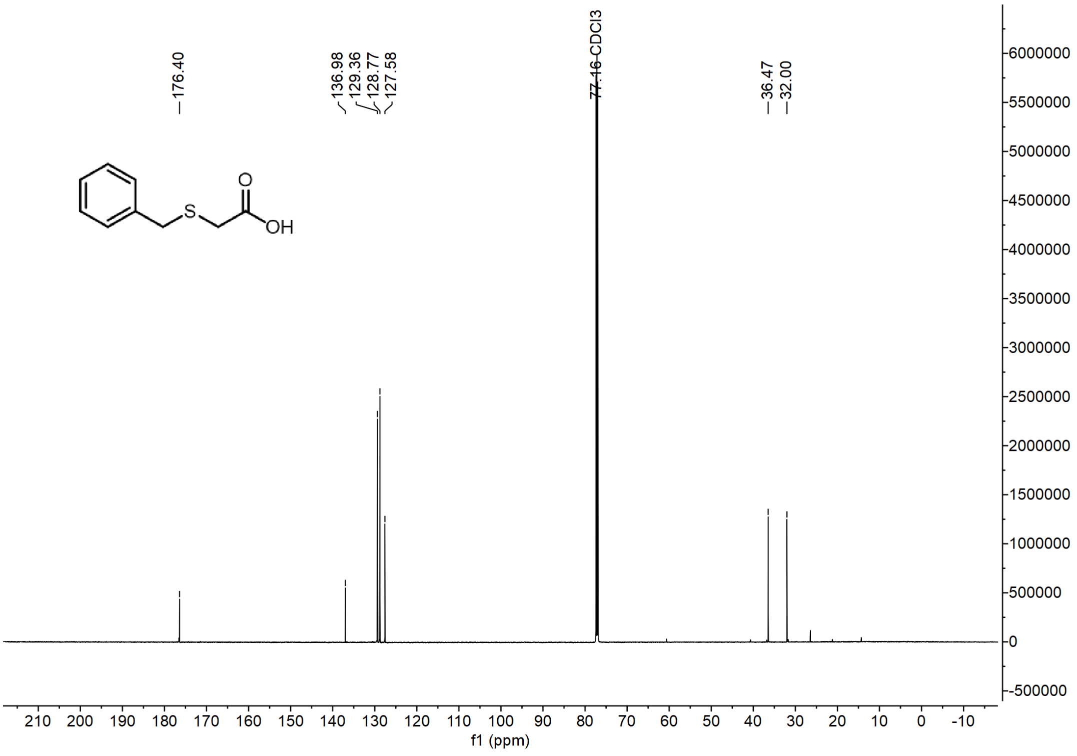

Supplementary Fig. 19 | NMR Spectra of 2-(benzylthio)acetic acid

S-benzyl-NHS-ester (^{1}H NMR 601 MHz, $CDCl_3$)

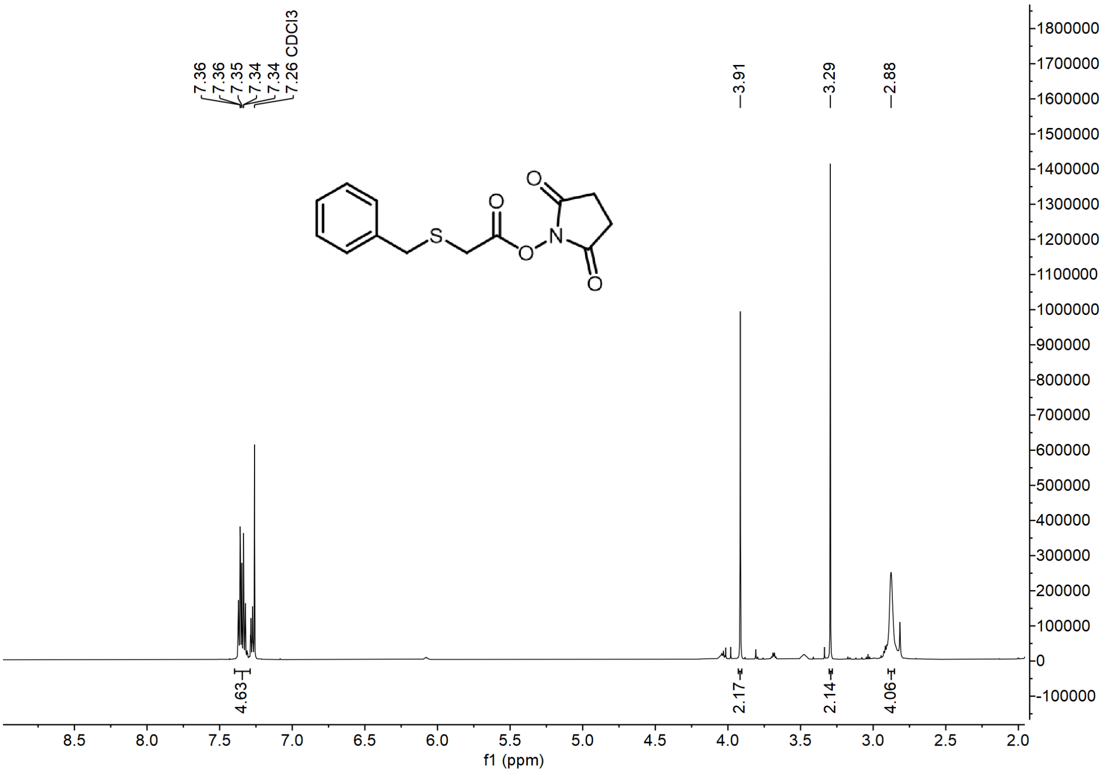

S-benzyl-NHS-ester (^{13}C NMR 151 MHz, CDCl3)

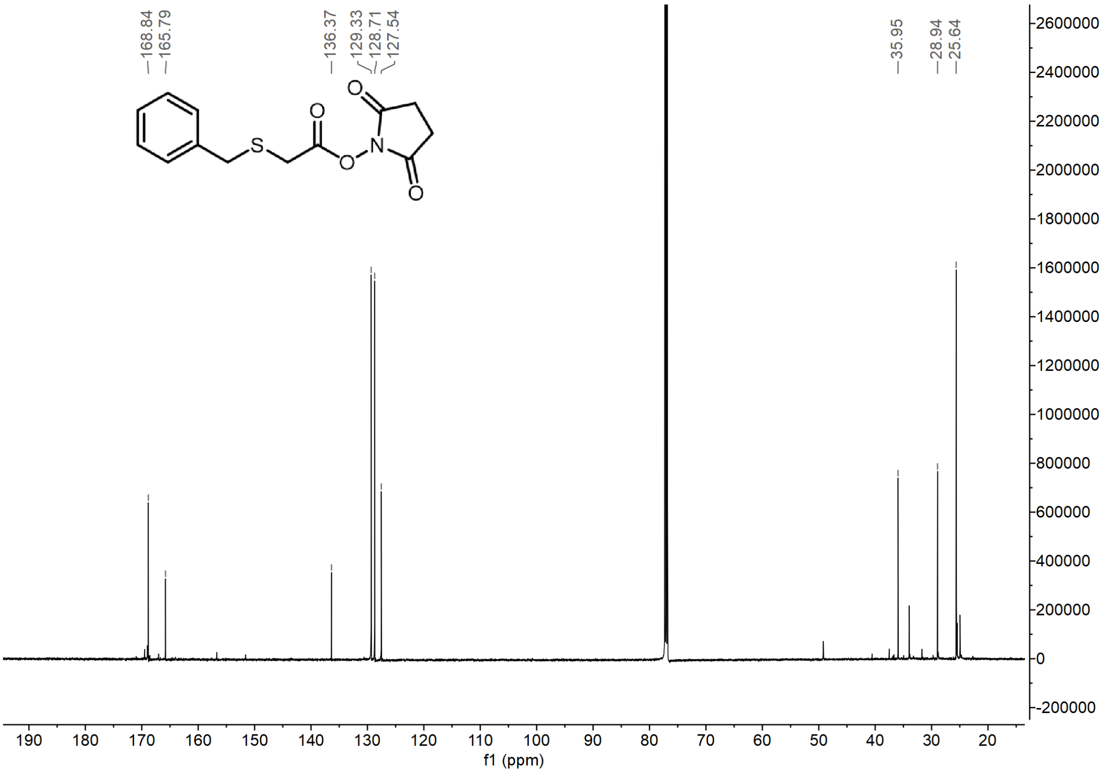

Supplementary Fig. 20 | NMR Spectra of S-benzyl-NHS-ester

benzyl 2-mercaptoacetate (^{1}H NMR 400 MHz, $CDCl_3$)

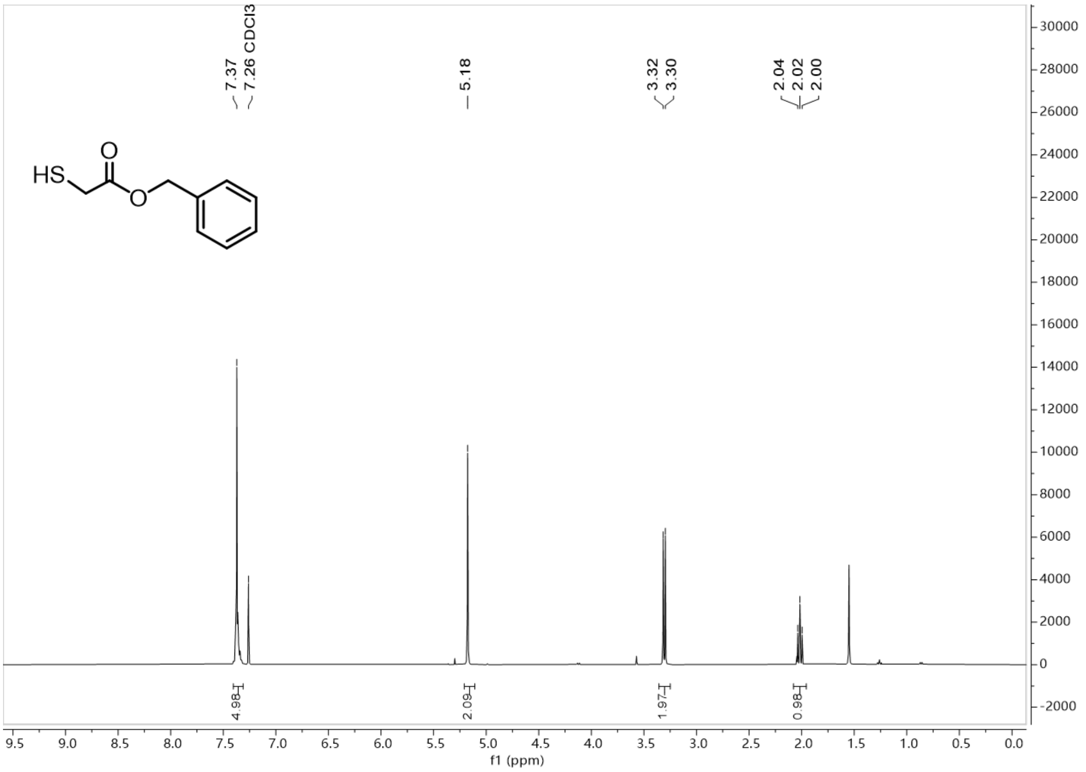

Supplementary Fig. 21 | NMR Spectra of benzyl 2-mercaptoacetate

Phenylmethyl 2-[(β-D-glucopyranosyl)thio]acetate (^{1}H NMR 400 MHz, D_2O)

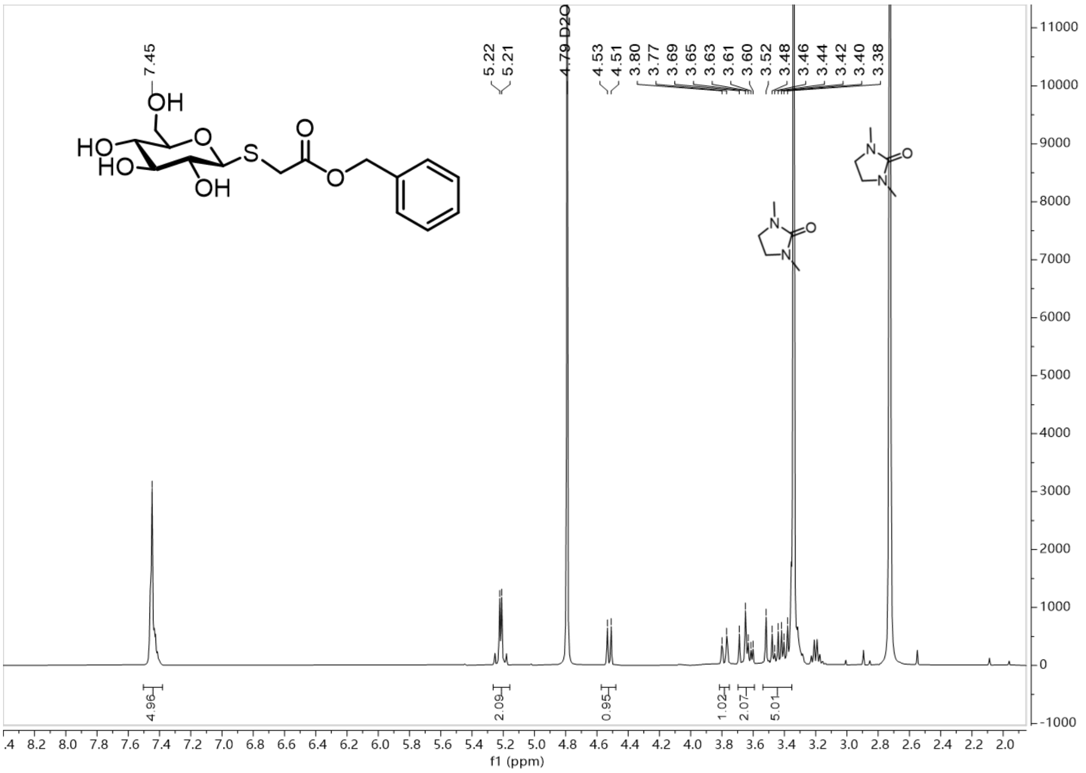

Phenylmethyl 2-[(β-D-glucopyranosyl)thio]acetate (^{13}C NMR 101 MHz, D_2O)

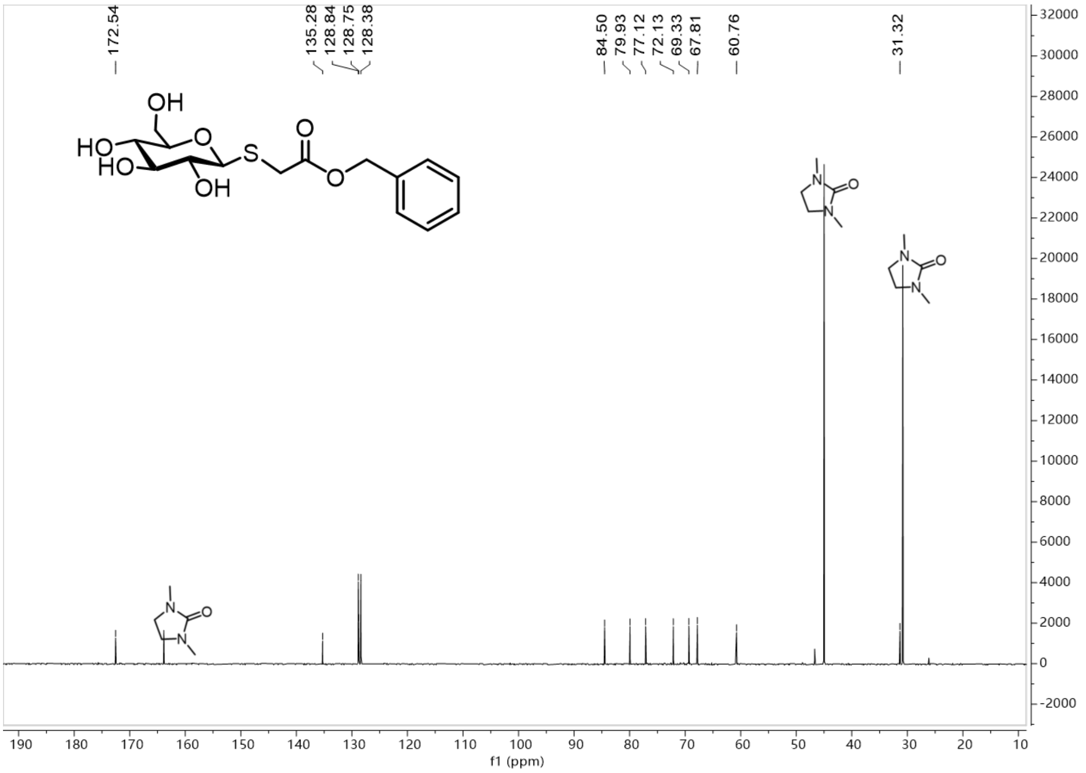

Phenylmethyl 2-[(β-D-glucopyranosyl)thio]acetate (HSQC, D_2O)

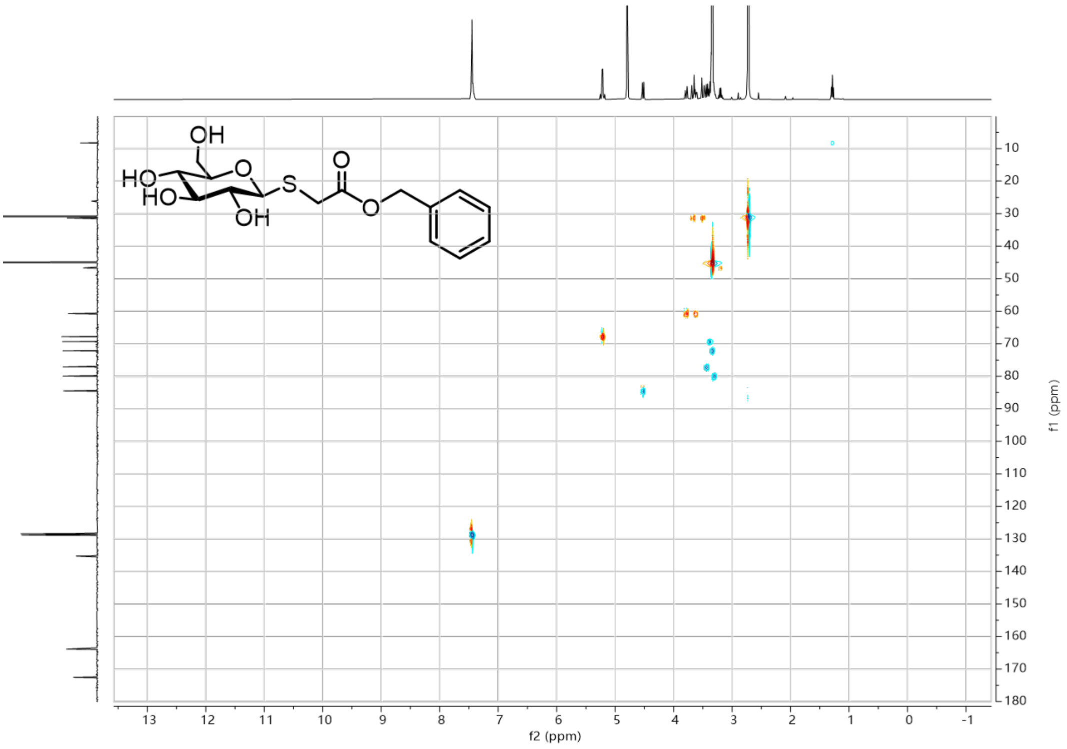

Phenylmethyl 2-[(β-D-glucopyranosyl)thio]acetate (HMBC, D_2O)

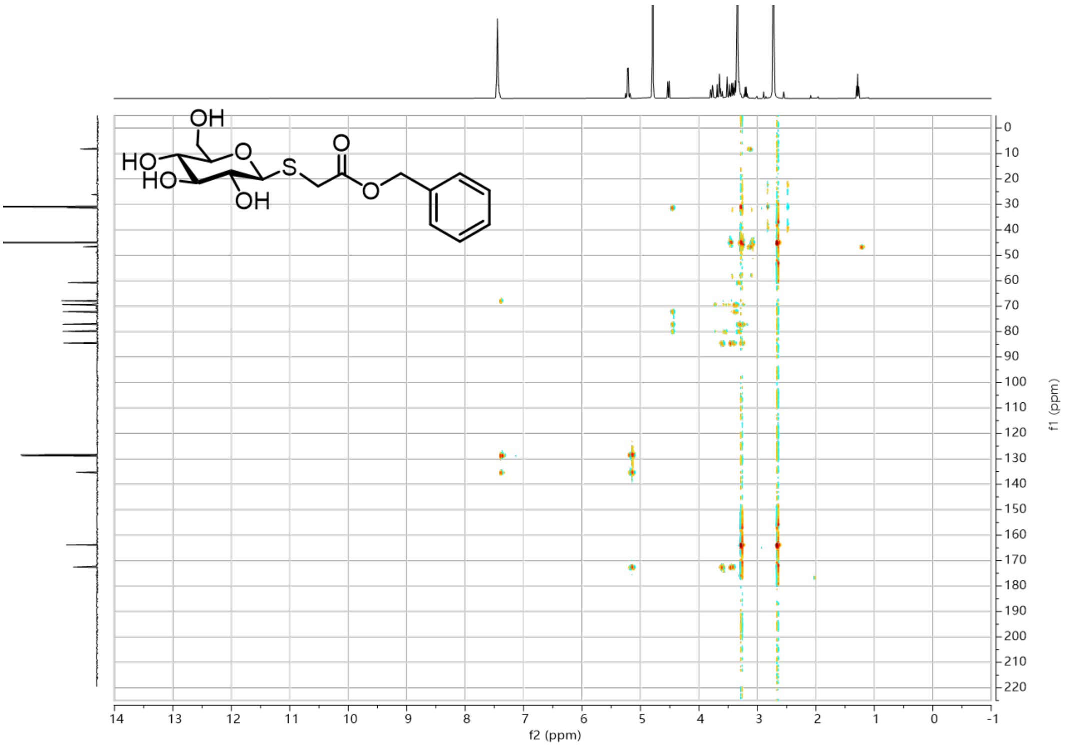

Supplementary Fig. 22 | NMR Spectra of Phenylmethyl 2-[(β-D-glucopyranosyl)thio]acetate

β-D-Glucosidothioglycolic acid (^{1}H NMR 400 MHz, D_2O)

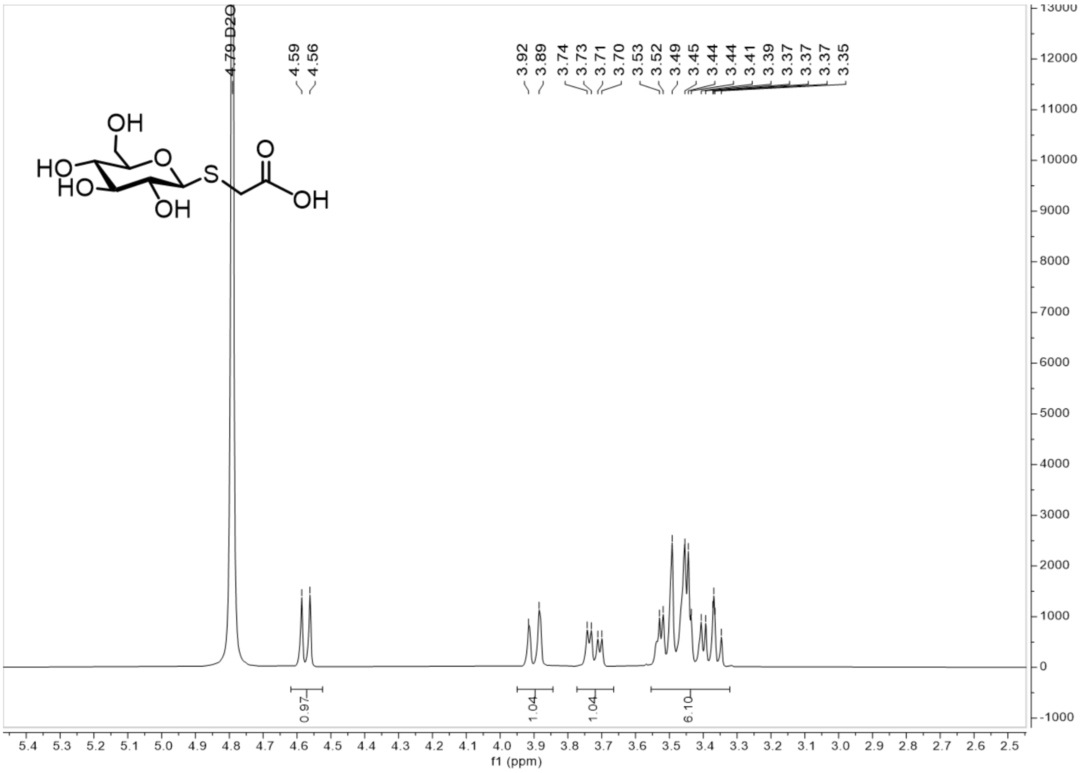

β-D-Glucosidothioglycolic acid (^{13}C NMR 101 MHz, D_2O)

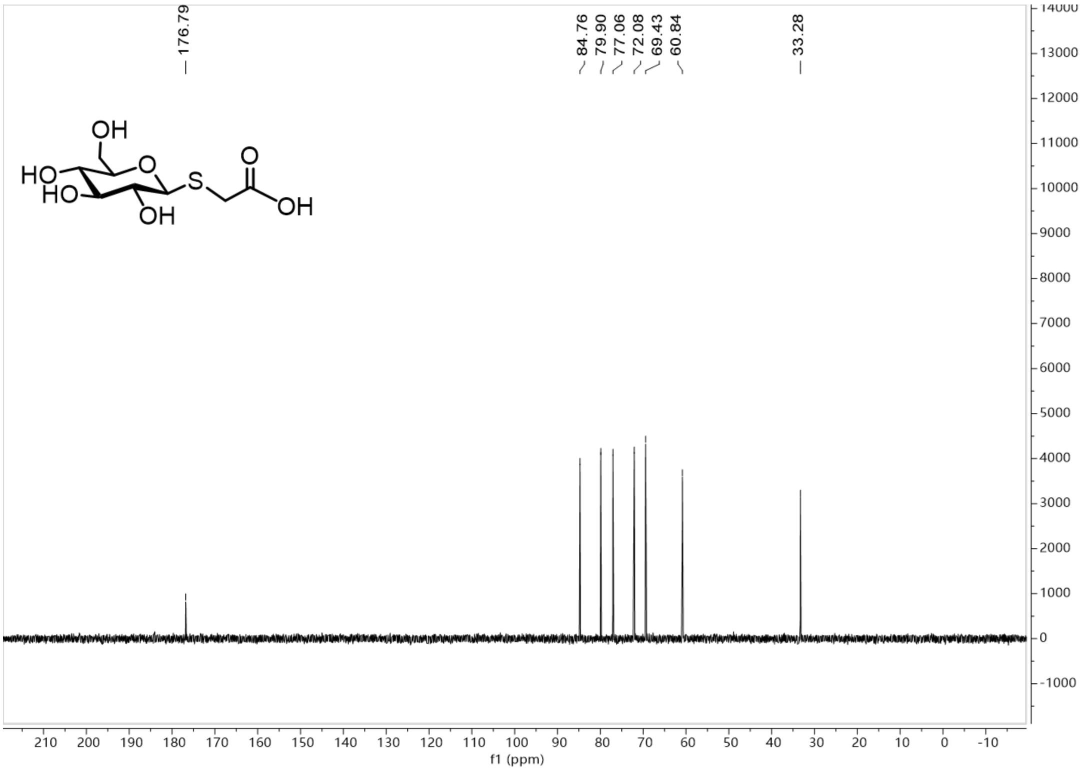

β-D-Glucosidothioglycolic acid (HSQC, D_2O)

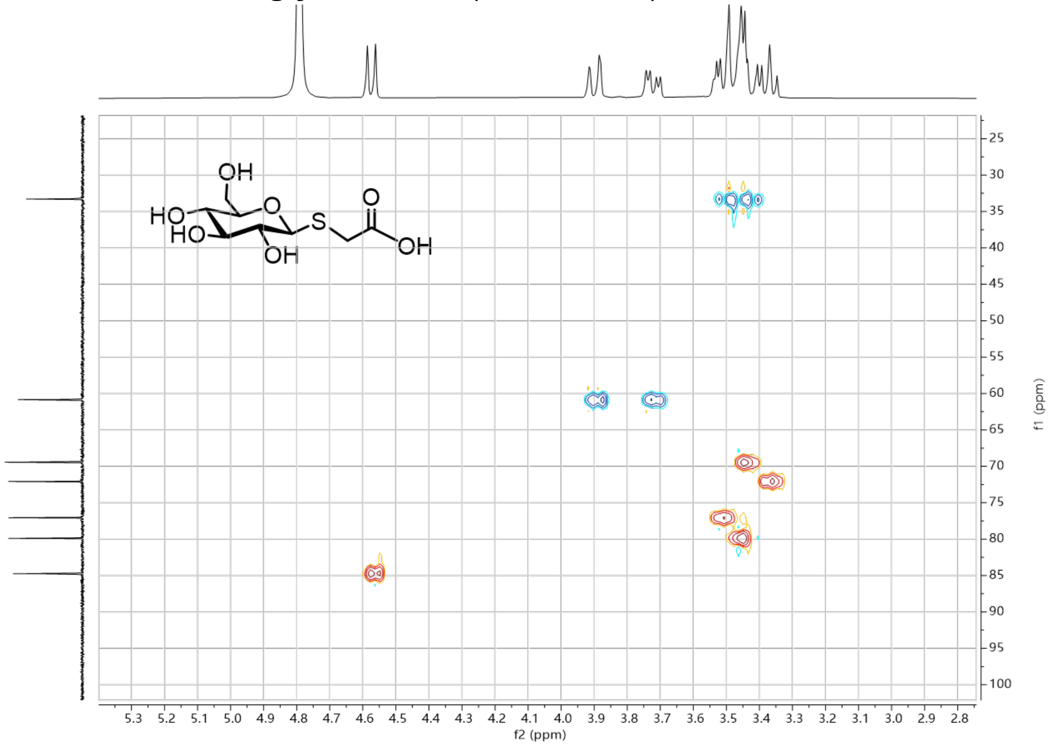

β-D-Glucosidothioglycolic acid (HMBC, D_2O)

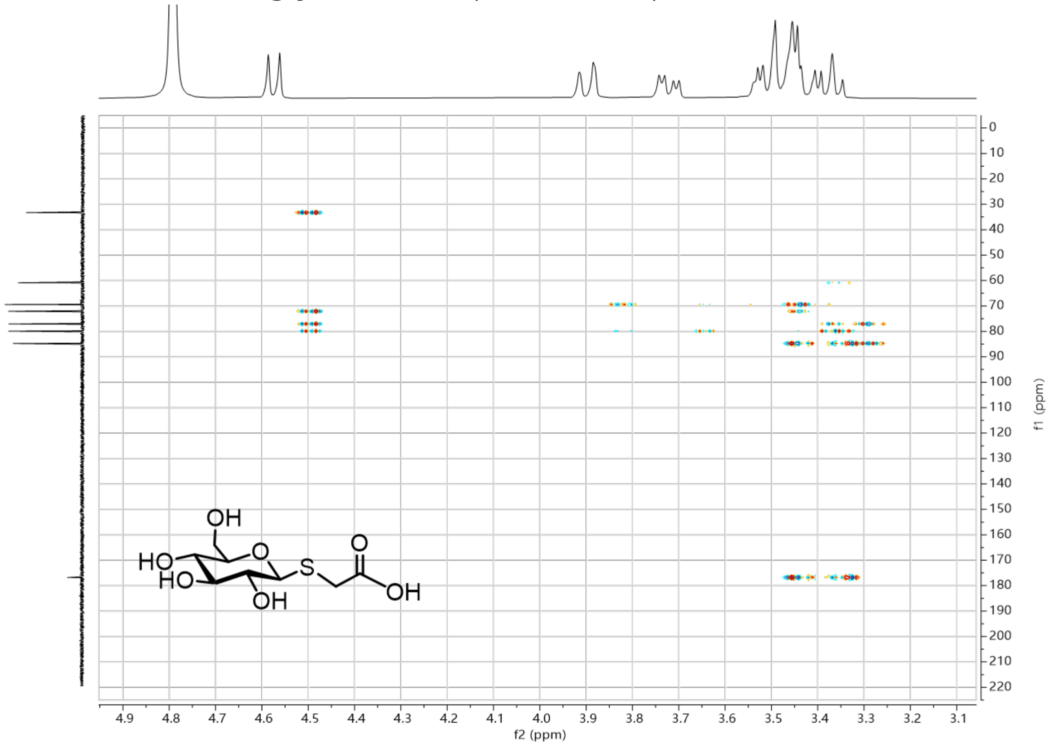

Supplementary Fig. 23 | NMR Spectra of β-D-Glucosidothioglycolic acid

Glc-NHS (^1H NMR 400 MHz, D_2O)

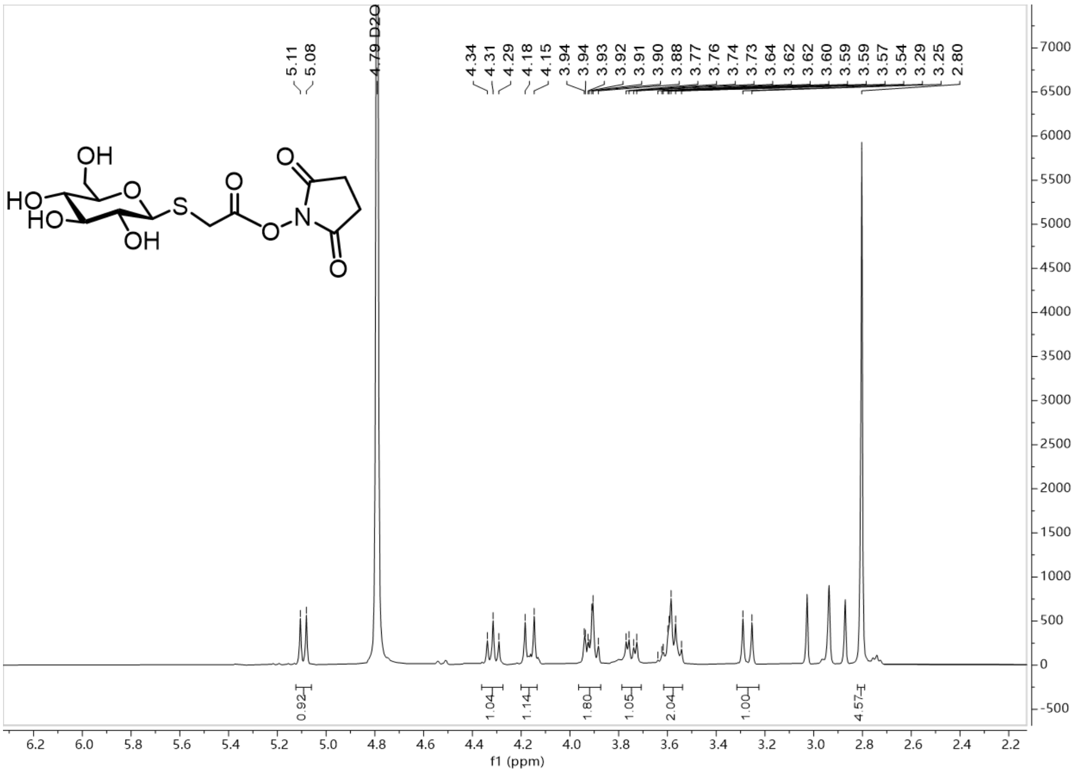

Glc-NHS (^{13}C NMR 101 MHz, D_2O)

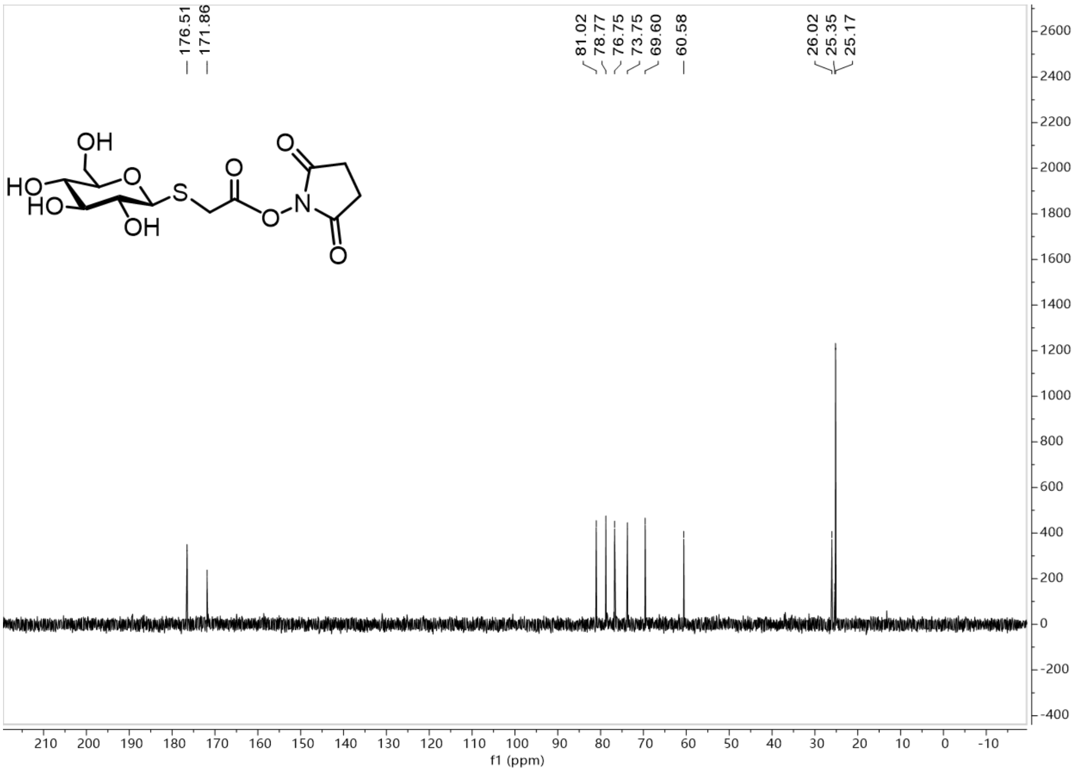

Glc-NHS (COSY, D_2O)

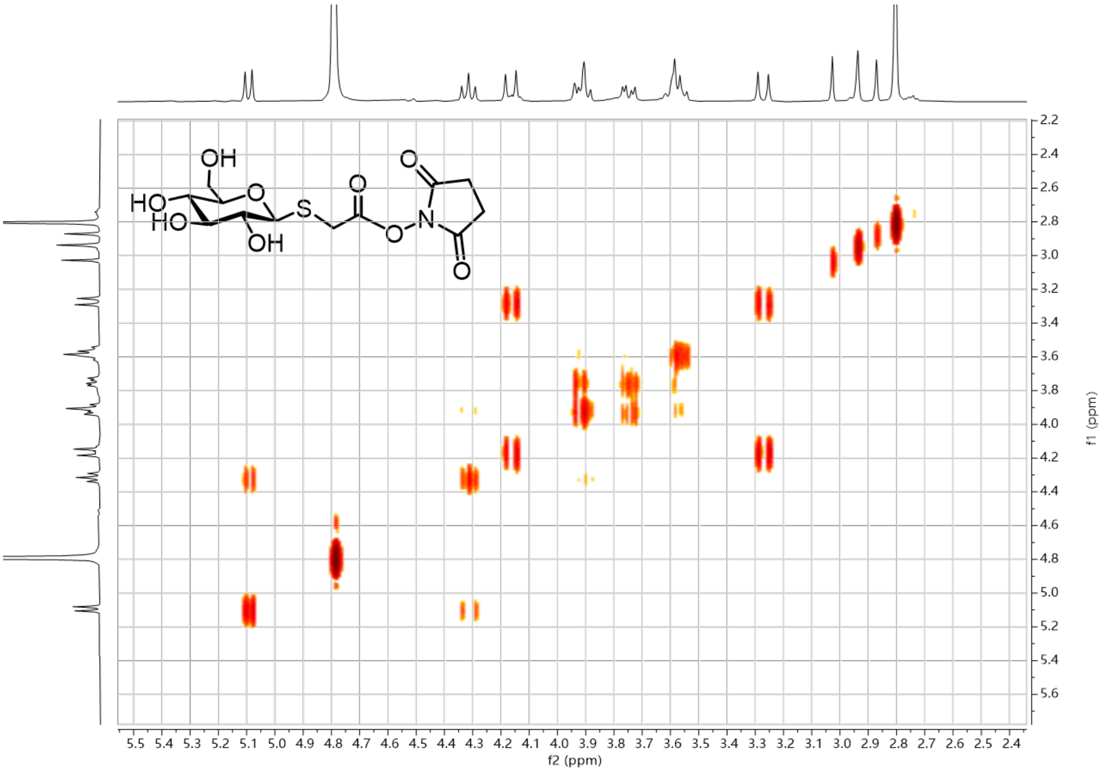

Glc-NHS (HSQC-NMR, D_2O)

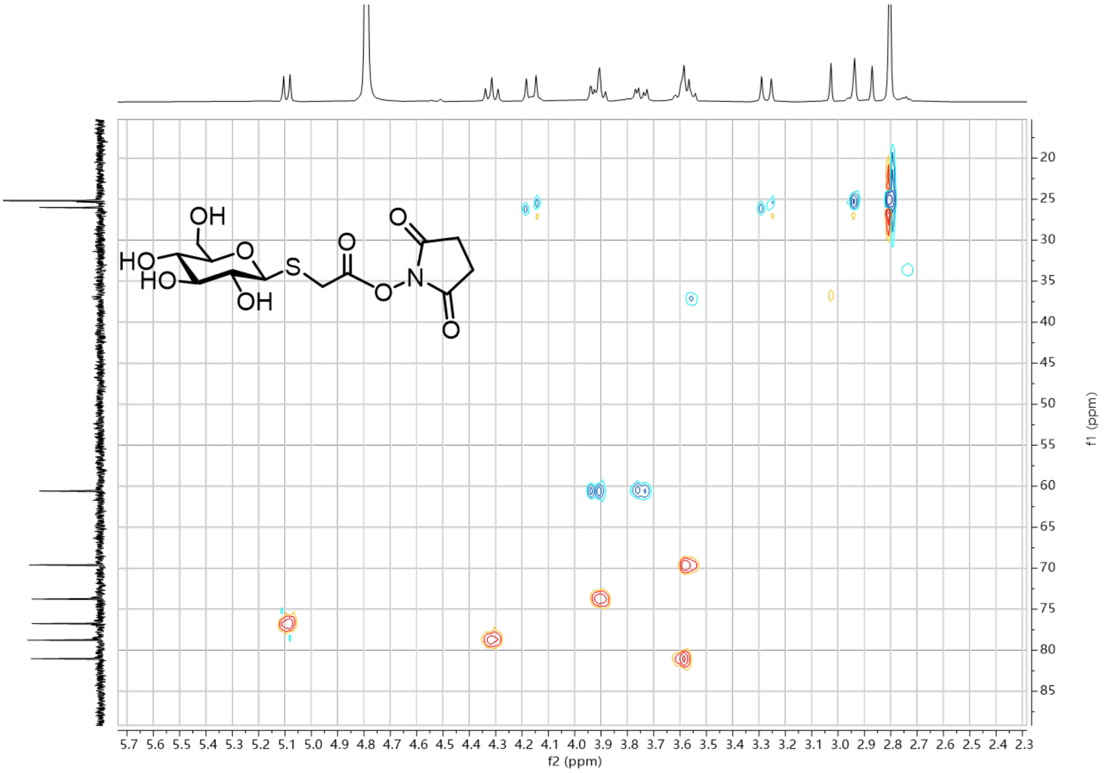

Glc-NHS (HMBC-NMR, D_2O)

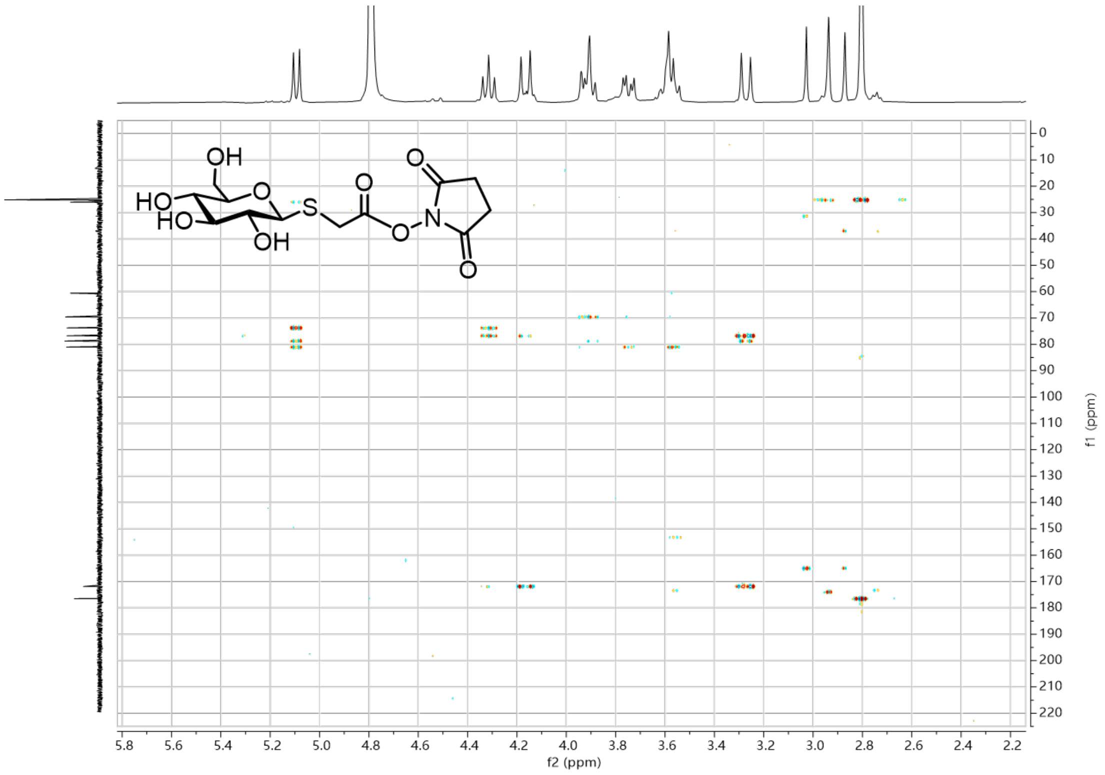

Supplementary Fig. 24 | NMR Spectra of Glc-NHS

Ac-Lac-SAc (^{1}H NMR 400 MHz, $CDCl_3$)

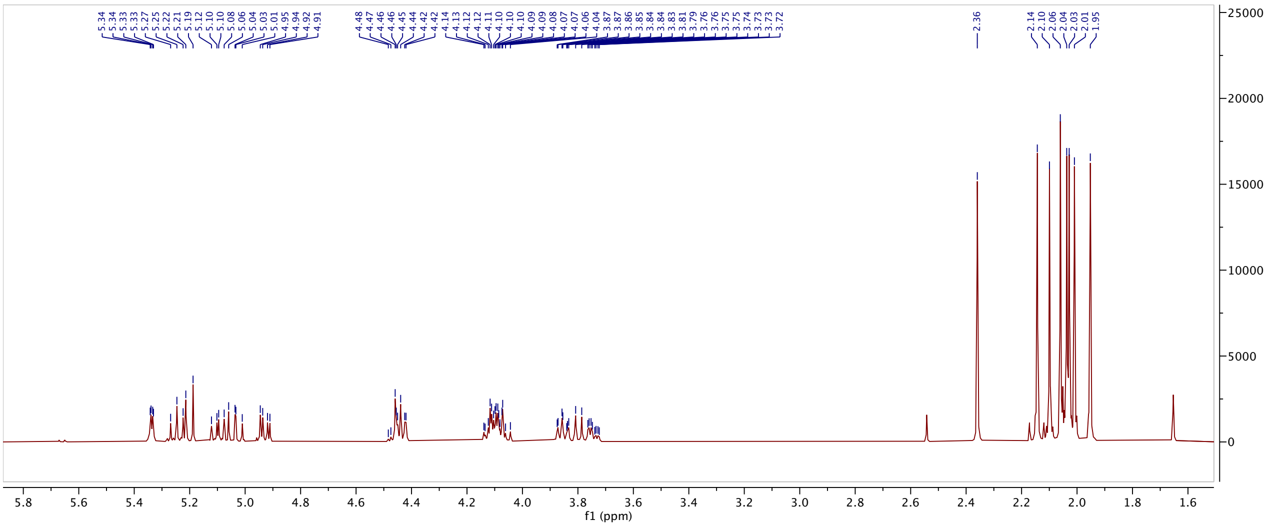

Ac-Lac-SAc (^{13}C NMR 101 MHz, $CDCl_3$)

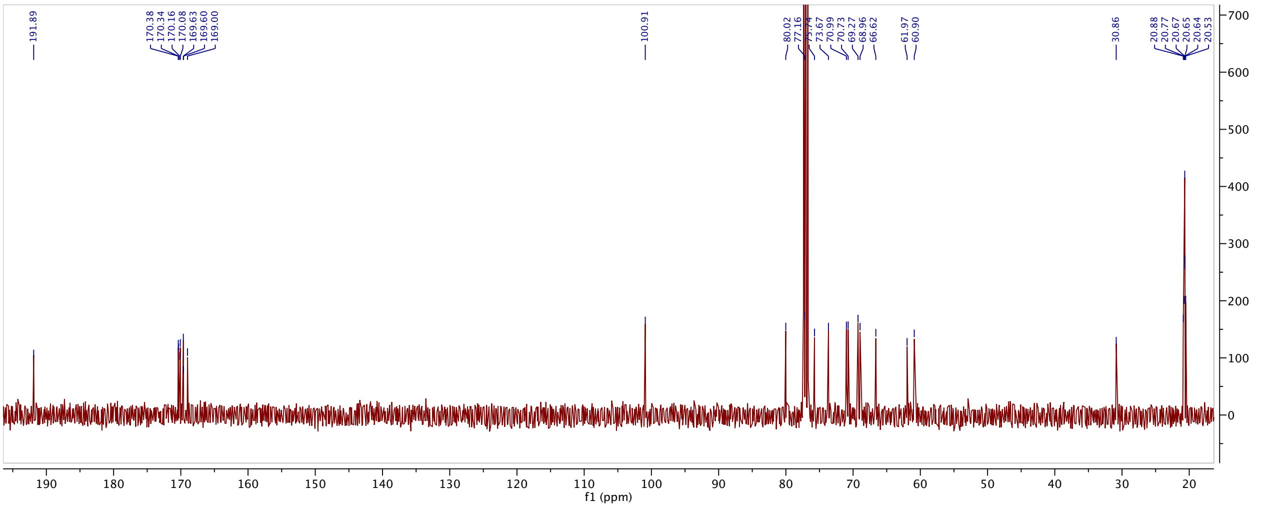

Supplementary Fig. 25 | NMR Spectra of Ac-Lac-SAc

Ac-Lac-SCH2COOH (^{1}H NMR 400 MHz, $CDCl_3$)

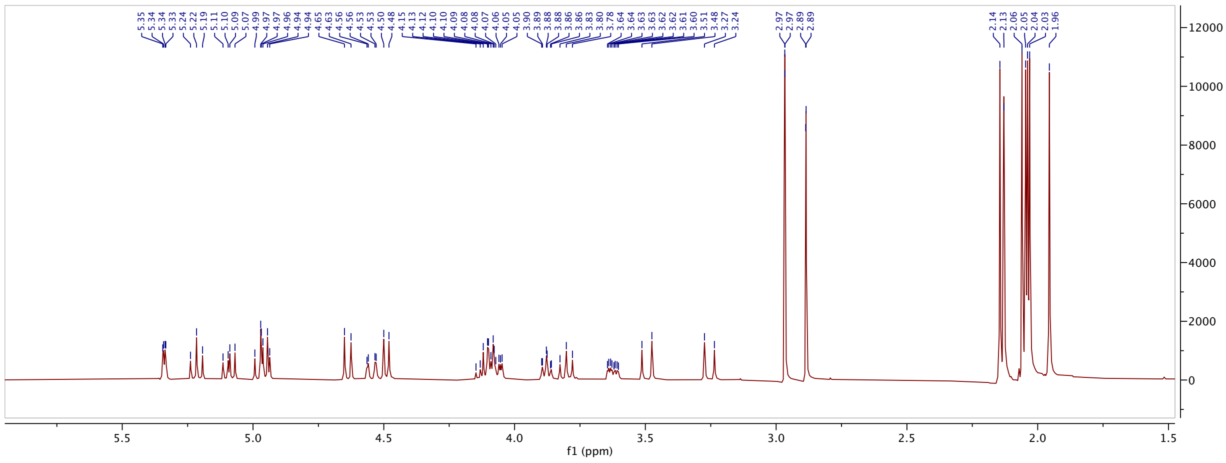

Ac-Lac-SCH2COOH (^{13}C NMR 101 MHz, $CDCl_3$)

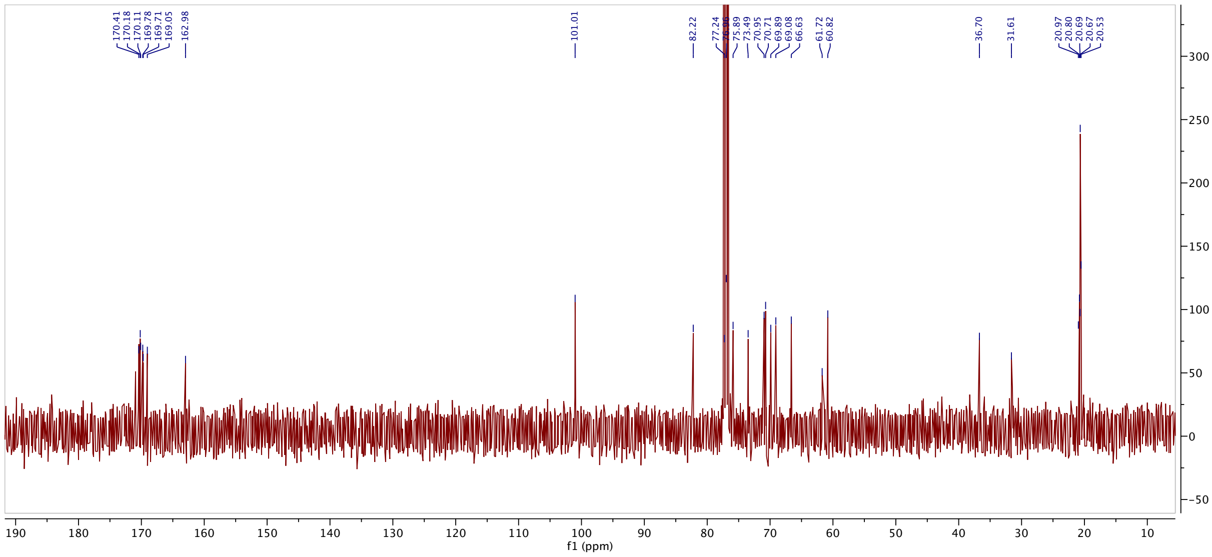

Supplementary Fig. 26 | NMR Spectra of Ac-Lac-SCH2COOH

Lac-SCH$_2$COOH (^{1}H NMR 400 MHz, D_2O)

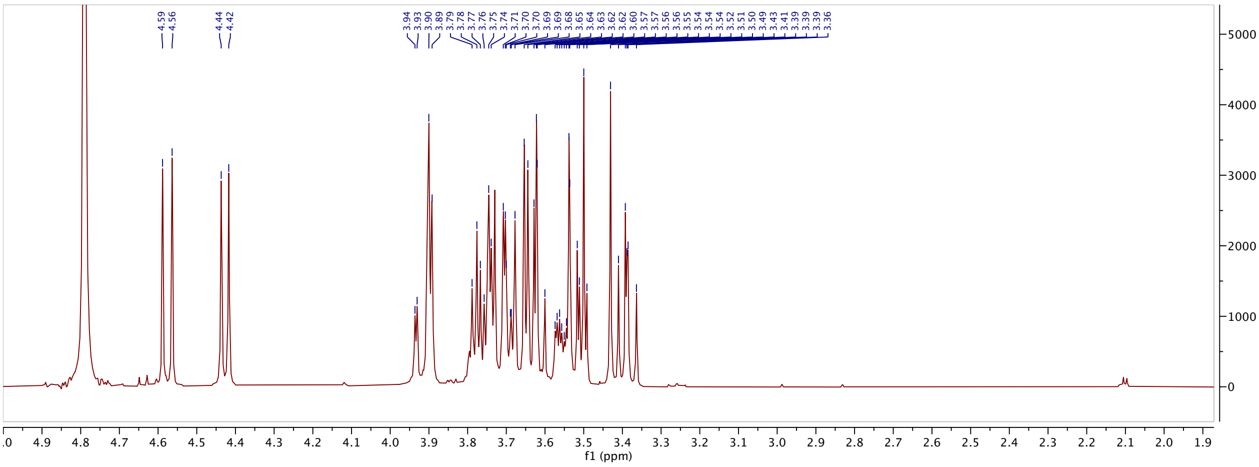

Lac-SCH$_2$COOH (^{13}C NMR 101 MHz, D_2O)

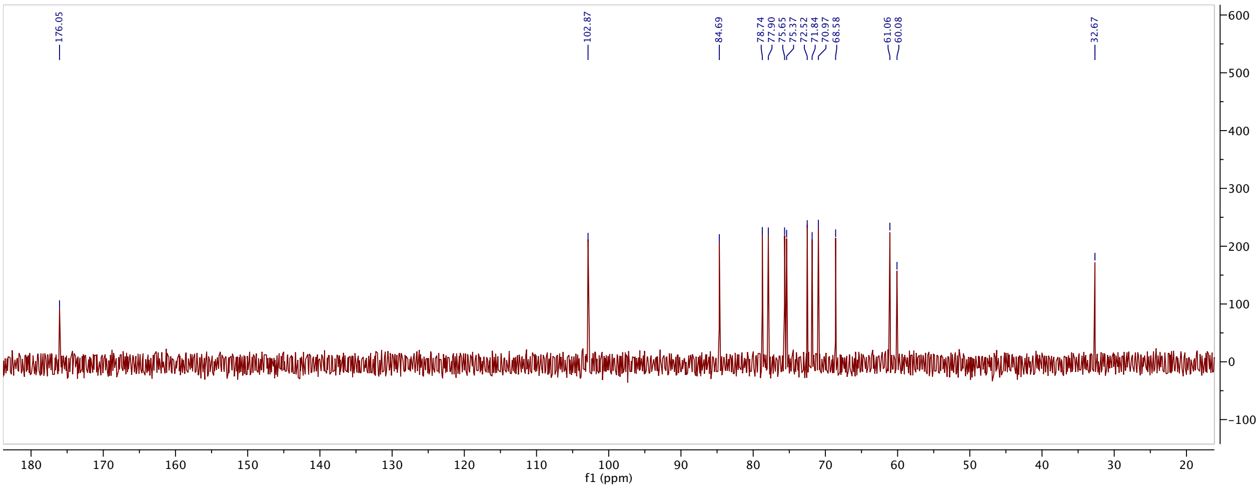

Supplementary Fig. 27 | NMR Spectra of Lac-SCH$_2$COOH

Gal-Glc-NHS (^{1}H NMR 400 MHz, D_2O)

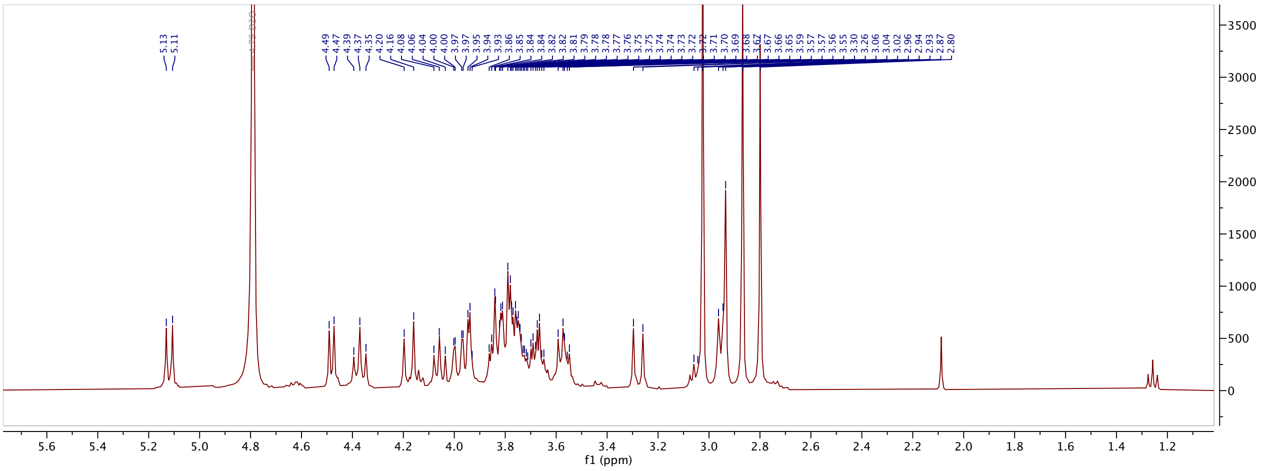

Gal-Glc-NHS (^{13}C NMR 101 MHz, D_2O)

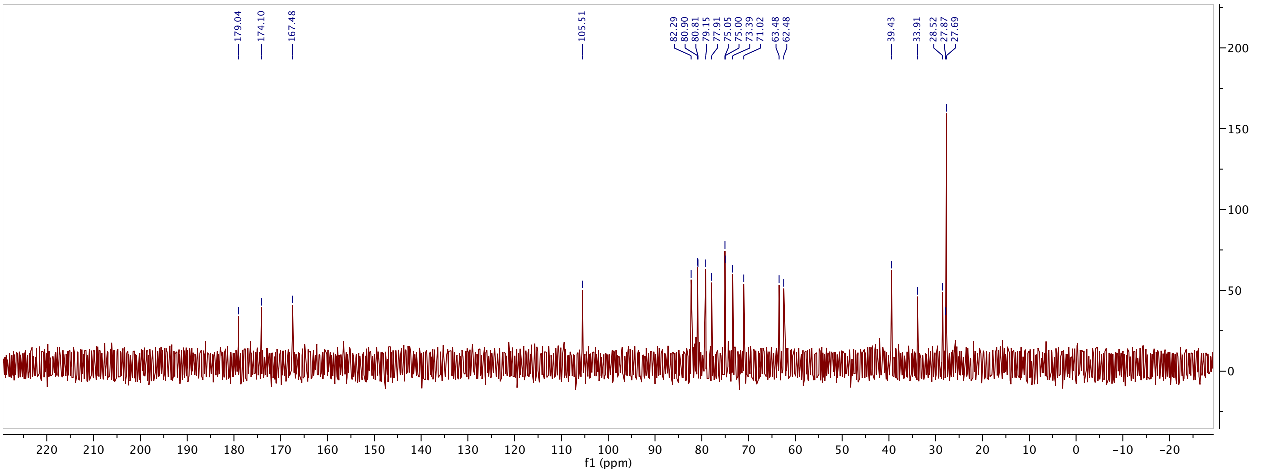

Gal-Glc-NHS (HSQC-NMR, D_2O)

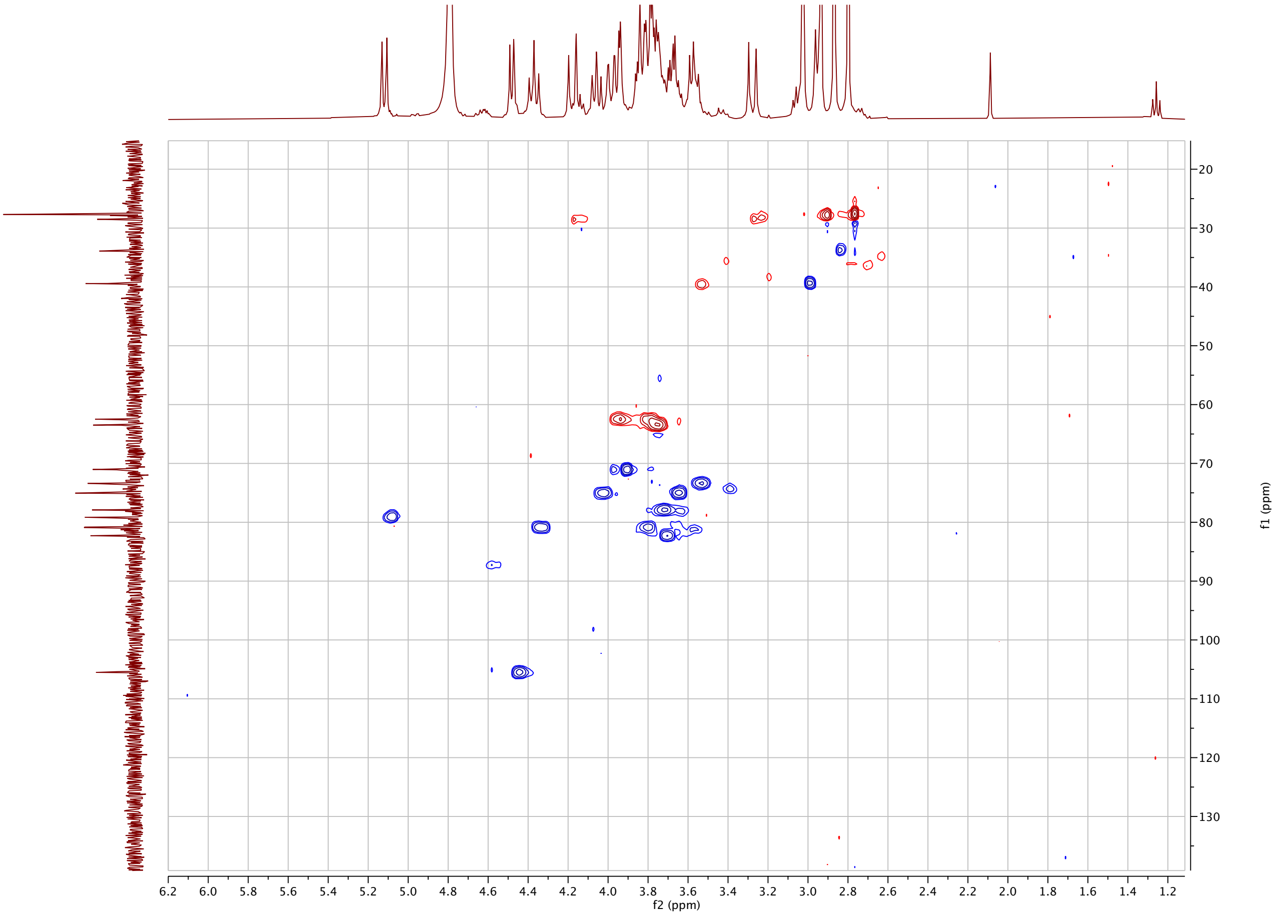

Supplementary Fig. 28 | NMR Spectra of Gal-Glc-NHS

Sia-Gal-Glc-SCH₂COOH (^{1}H NMR 400 MHz, D_2O)

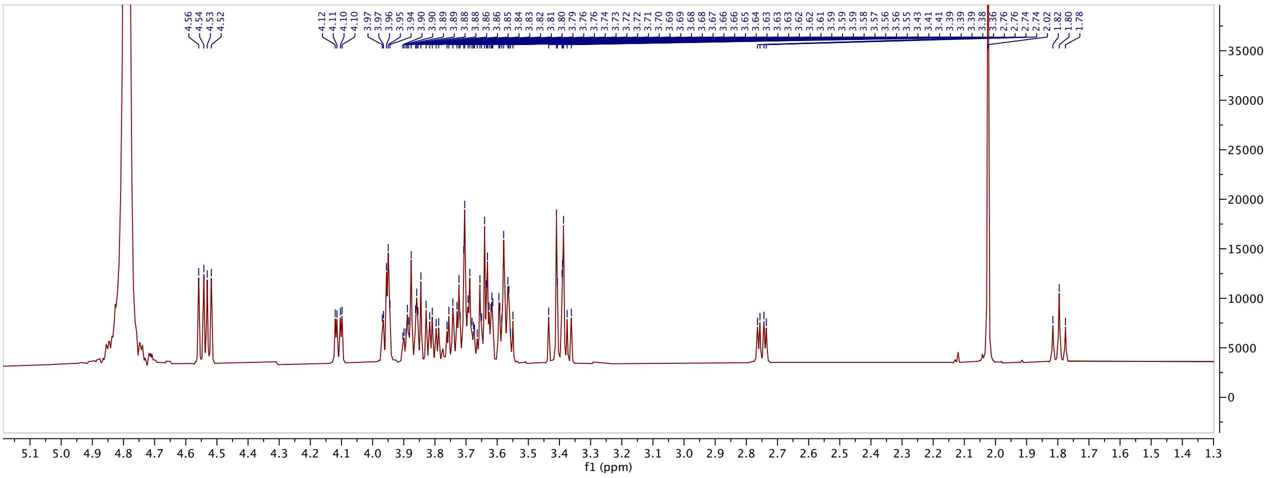

Sia-Gal-Glc-SCH₂COOH (^{13}C NMR 101 MHz, D_2O)

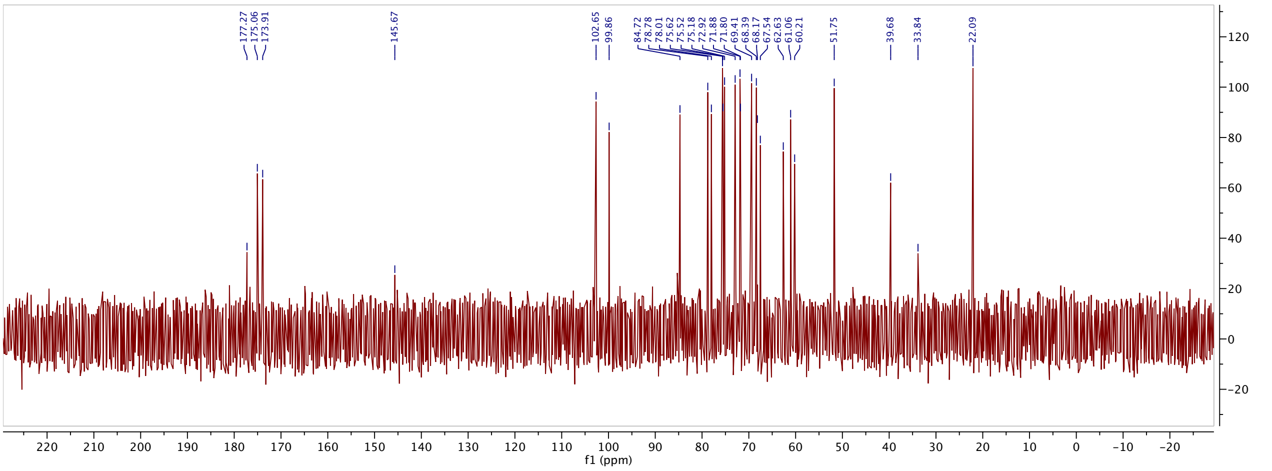

Sia-Gal-Glc-SCH$_2$COOH (HSQC-NMR, D$_2$O)

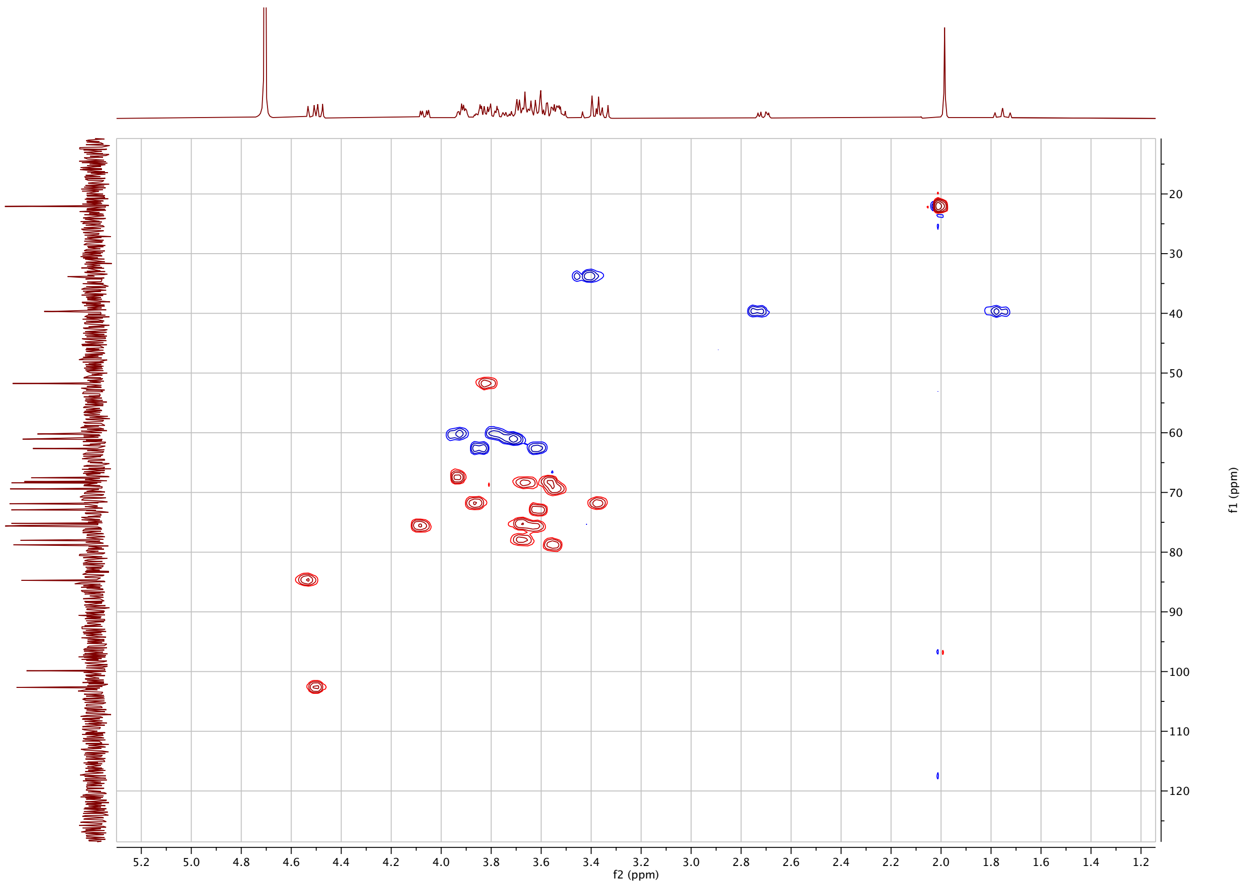

Supplementary Fig. 29 | NMR Spectra of Sia-Gal-Glc-SCH$_2$COOH

Sia-Gal-Glc-NHS (^1H NMR 400 MHz, D_2O)

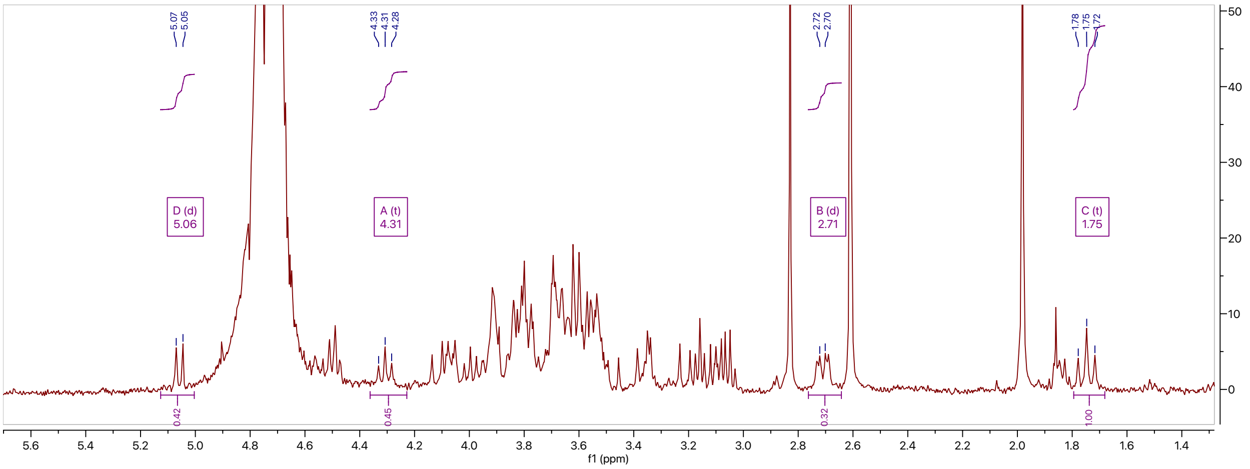

Sia-Gal-Glc-NHSS (^1H NMR 400 MHz, D_2O)

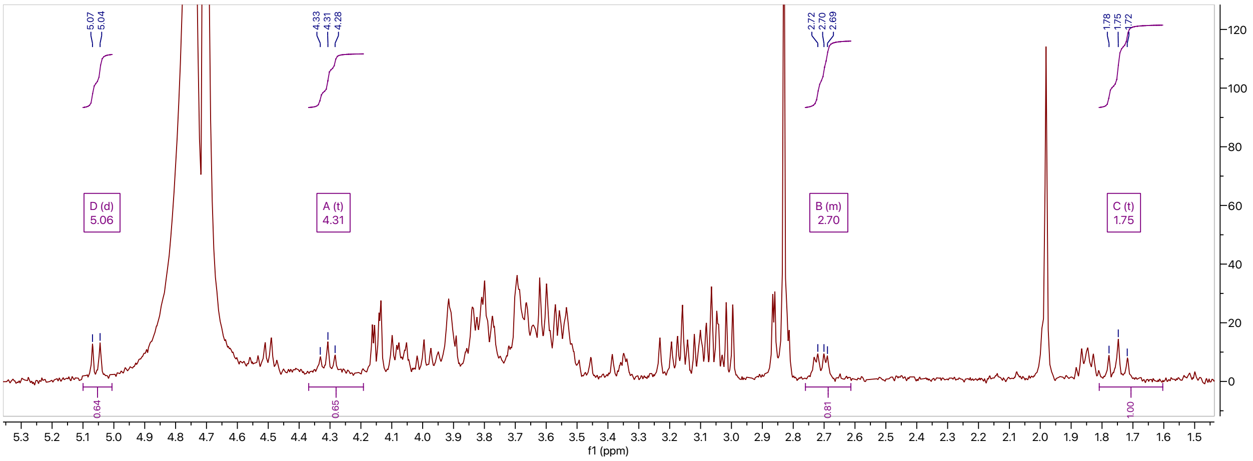

Sia-Gal-Glc-PFP (^1H NMR 400 MHz, D_2O)

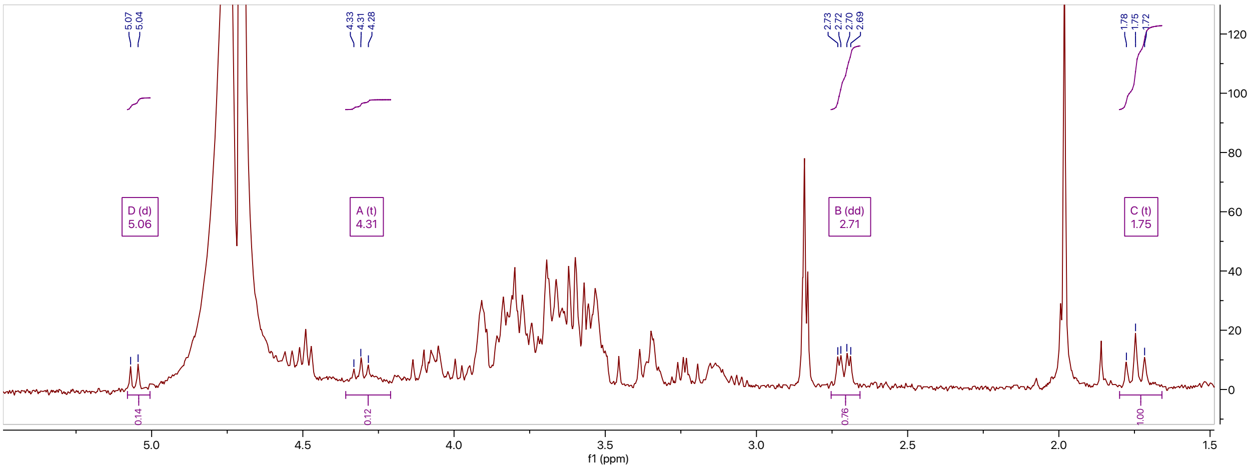

Supplementary Fig. 30 | NMR Spectra of Activated Sia-Gal-Glc Esters

2-(Acetylthio)acetic acid (^{1}H NMR 601 MHz, $CDCl_3$)

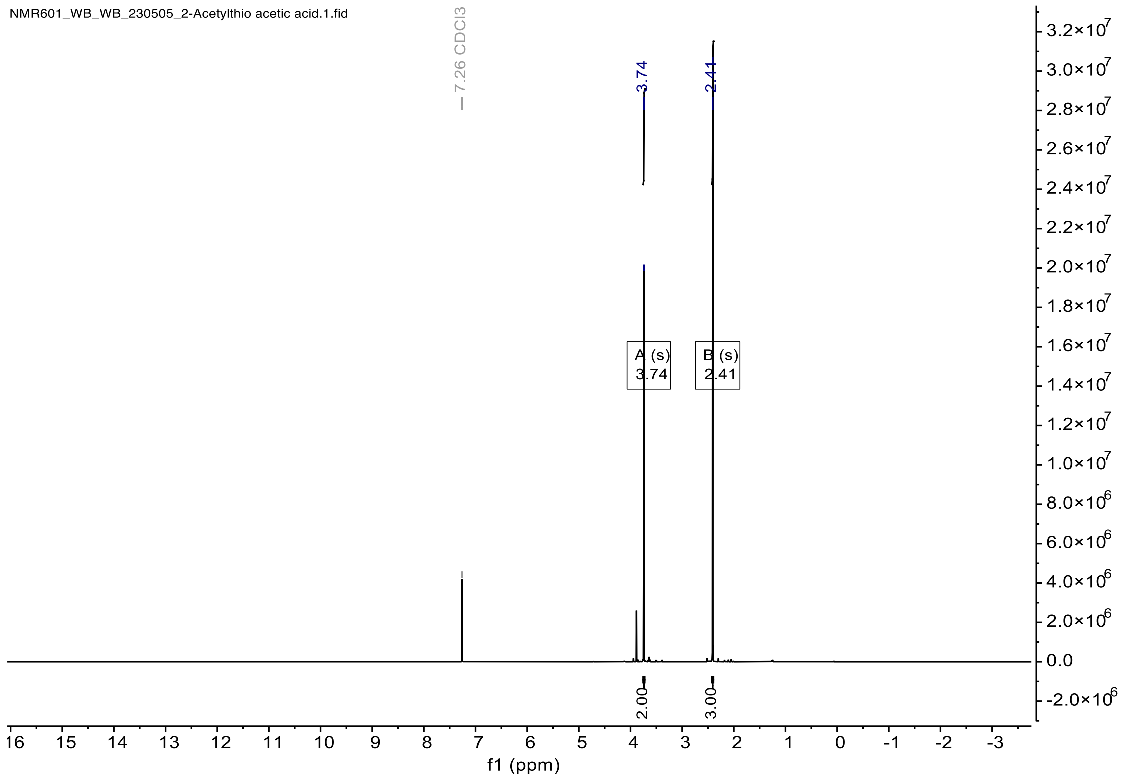

2-(Acetylthio)acetic acid (^{13}C NMR 151 MHz, $CDCl_3$)

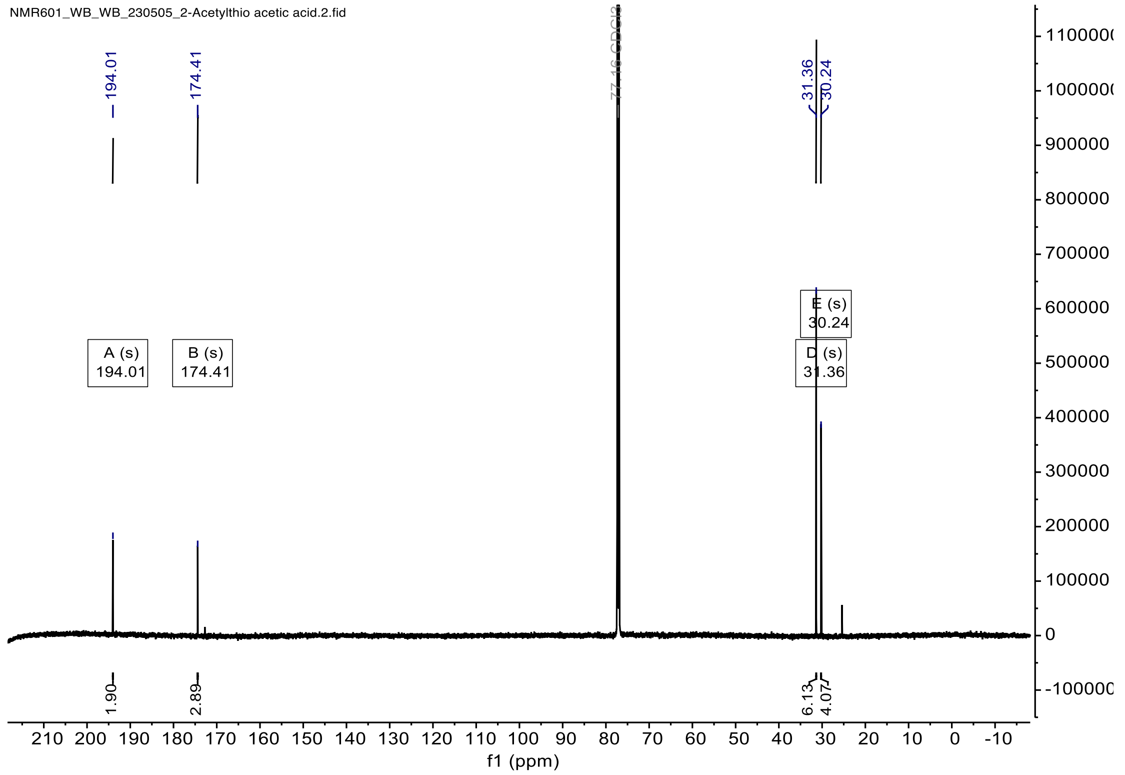

2-(Acetylthio)acetic acid (HSQC, $CDCl_3$)

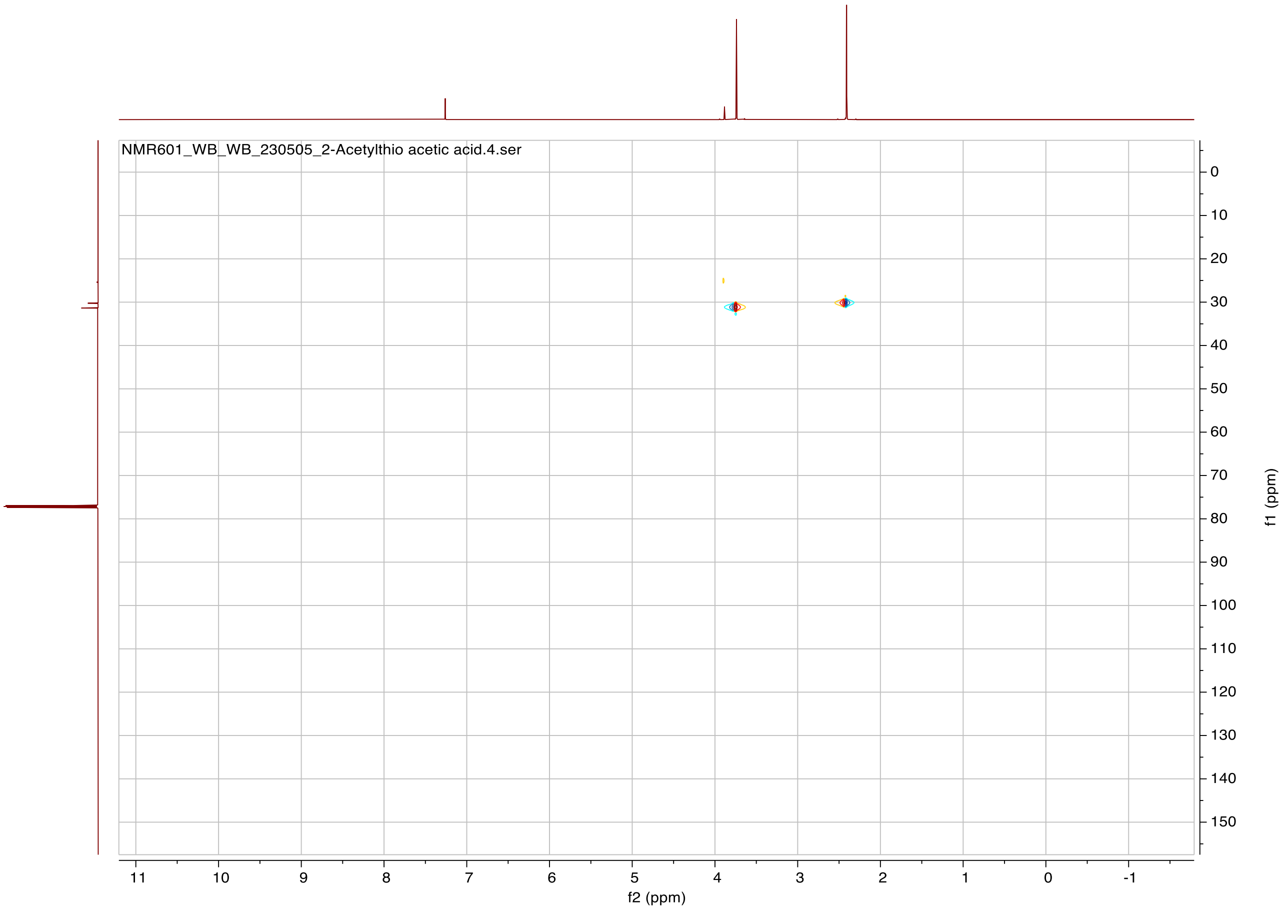

Supplementary Fig. 31 | NMR Spectra of 2-(Acetylthio)acetic acid

SATA-NHSS (^{1}H NMR 400 MHz, DMSO-d6)

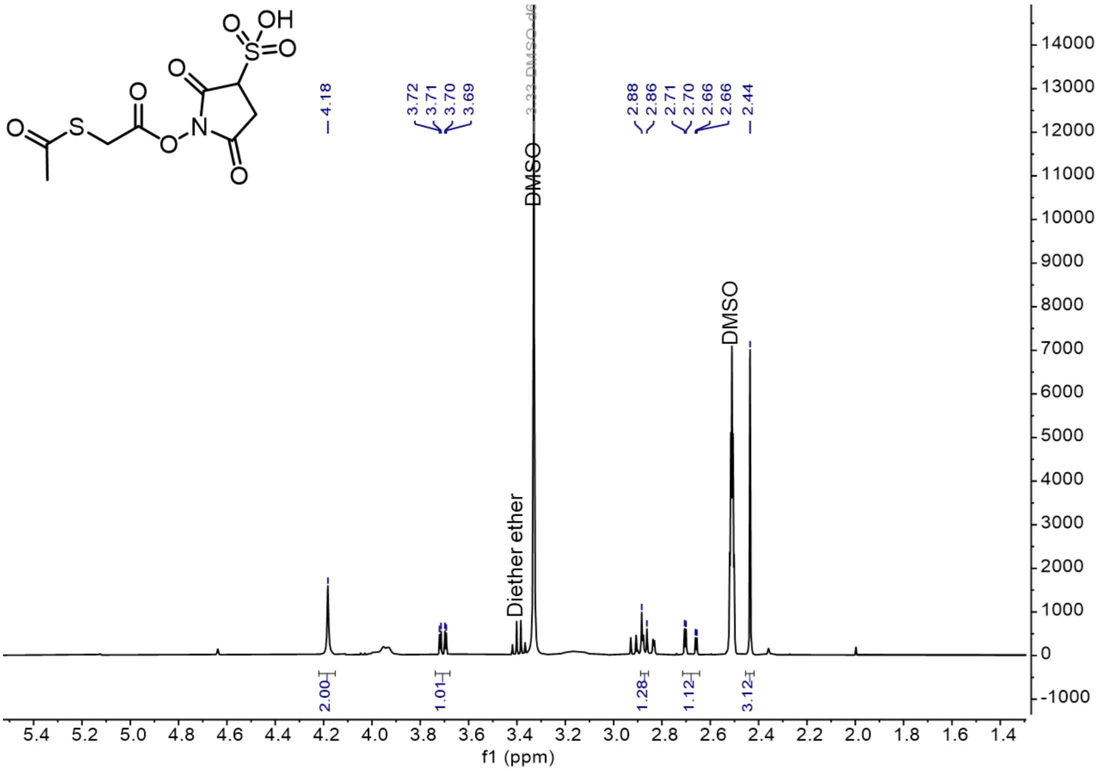

SATA-NHSS (^{13}C NMR 101 MHz, DMSO-d6)

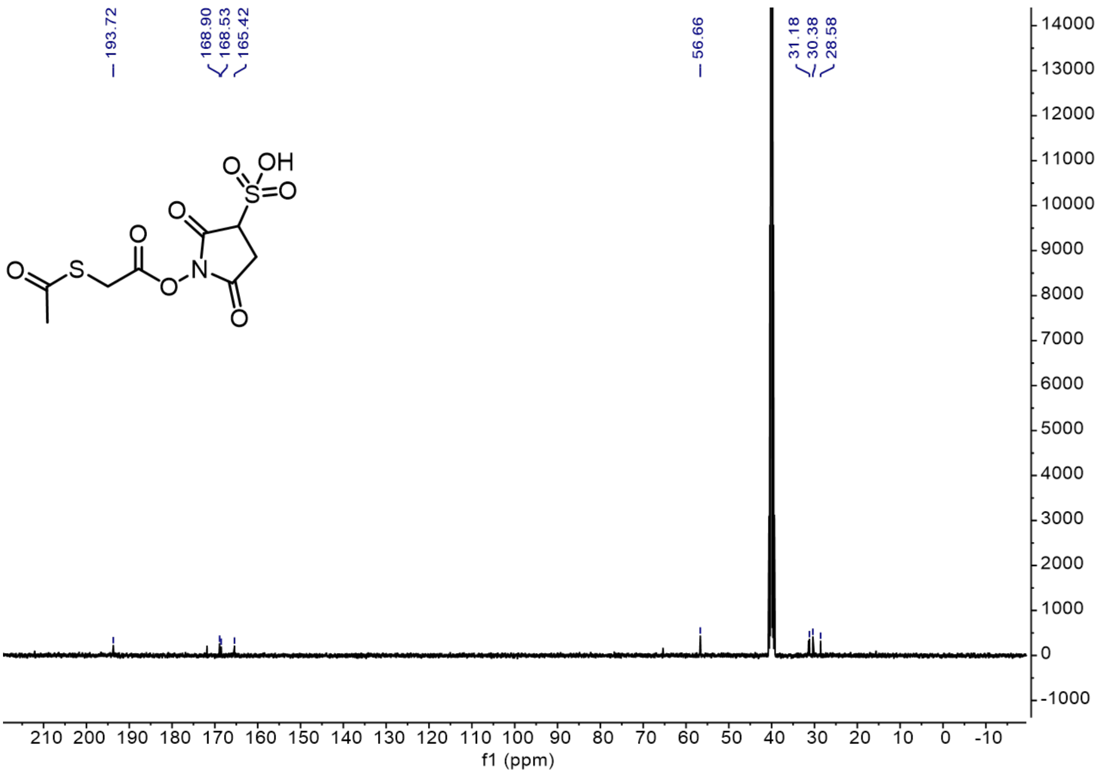

Supplementary Fig. 32 | NMR Spectra of SATA-NHSS

Supplementary Tables

Supplementary Table 1 | Data analysis parameters. Exact expected mass difference for each of the modifications for reagents that were tested in this study.

Substrates	*N*-acyl derivatives	*N*-succinamide derivatives	acetamides	NHS-scar
Sia-Gal-Glc-NHS	689.1831	804.2099	-	115.0272
Gal-Glc-NHS	398.0869	513.1152	-	115.0272
Glc-NHS	236.0354	351.0624	-	115.0272
S-benzyl-NHS-ester	164.0302	279.0571	-	115.0272
SATA	115.9932/131.0038*	230.0123	42.0106	115.0272
SATP	130.0089/145.0188*	245.0358	42.0106	115.0272
Sia-Gal-Glc-NHSS	689.1831	884.1680	-	194.9843
SATA-NHSS	115.9932/131.0038*	310.9775	42.0106	194.9843
Sia-Gal-Glc-S-PFP	689.1831	-	-	-

*For MS/MS analysis of SATA, SATP, and SATA-NHSS, +15.0109 (artefacts due to sample preparation) for *N*-acyl derivatives might to be used.

Supplementary Table 2 | Intact protein chromatography parameters.

Step	Time / min	%Solvent A	%Solvent B
0	0	95	5
1	1.0	95	5
2	7.0	5	95
3	8.0	5	95
4	8.1	95	5
5	10.0	95	5

Supplementary Experimental Procedures

Reagent and materials

Unless otherwise noted, the chemicals and solvents used were of analytical grade and were used as received from commercial sources. Lysozyme from hen egg white (lyophilized powder, ≥40,000 units/mg protein, Cat#L6876), DL-Dithiothreitol (Cat#D9779), SATA (≥95%, Cat#A9043), SATP (≥95%, Cat#10859), Fmoc *N*-hydroxysuccinimide ester (≥98%, Cat#46920), Succinimidyl 4-(*N*-maleimidomethyl)cyclohexane-1-carboxylate (SMCC, ≥97%, Cat#M5525), Sulfo-SMCC (Cat#M6035), Dibenzocyclooctyne-*N*-hydroxysuccinimidyl ester (DBCO-NHS ester, Cat#761524), Suberic acid bis(*N*-hydroxysuccinimide ester) (DSS, Cat#S1885), *Neisseria meningitides* CMP-sialic acid synthetase (Cat#C1999), *Pasteurella multocida* Sialyltransferase (Cat#S1951) were purchased from Sigma Aldrich.

General considerations

All chemical reactions were carried out under an inert atmosphere using argon or nitrogen gas. All glassware was heat-dried unless aqueous chemistry was involved. Thin layer chromatography (TLC) was carried out using Merck silica-aluminium plates, with UV light (254 nm) and potassium permanganate, anisaldehyde or H_2SO_4 for visualisation. Column chromatography was performed using Merck Geduran® Si 60 silica gel or a Biotage Flash Purification System with a Kinesis Telos column. Room temperature refers to 20-25 °C. NMR data was obtained using Bruker AVIIIHD 400 MHz, Bruker AVII 500 MHz machines, and Bruker Avance NEO 600 MHz machines. Reference values for residual solvents: ^{1}H NMR- 7.26 ($CDCl_3$), 4.79 (D_2O) 3.31 (MeOD- D4) ppm, ^{13}C NMR- O = 77.2 (CDCl3), 49.0 (MeOD- D4) ppm. Where appropriate, COSY, HSQC experiments were carried out to aid assignment. Coupling constants (J) are given in Hz and are uncorrected. NMR data was analysed using Mestrenova (V-11.0.1-17801).

Mass spectroscopy data was collected on Agilent 6120 Quadrupole spectrometer (ES), Waters LCT Premier (ES) instruments and a Xevo G2-S Q-ToF mass spectrometer coupled with a Waters Acquity UPLC system.

Chemical synthesis

Synthesis route of S-benzyl-NHS-ester (**3**), Glc-NHS (**4**), Gal-Glc-NHS (**5**), Sia-Gal-Glc-NHS (**6**), SATA-NHSS (**7**), Sia-Gal-Glc-NHSS (**8**), and Gal-Glc-S-PFP (**9**) refer to Supplementary Fig. 3, 4, 5, and 6.

2-(benzylthio)acetic acid ($C_9H_{10}O_2S$, 182.24 g/mol)

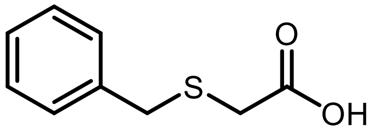

Synthesis of **2-(benzylthio)acetic acid** was carried out similar to literature procedure.[1] To a solution of benzyl bromide (0.171 g, 1 mmol) in dry methanol (3 mL) with NaOH (0.08 g, 2 mmol), thioglycolic acid (0.092g, 0.070 mL, 1 mmol) was dropwise added and stirred room temperature for 0.5 h. The solvent was removed under vacuum, add 10 mL ddH_2O and adjusted the pH by 1 M HCl to a final 4.0. The solution was washed with EtOAc (4 x 5 mL). The aqueous layer was lyophilised, pre-adsorbed onto silica, and purified by flash column chromatography (Petroleum ether: EtOAc, 1:2.5) to afford a white powder (127 mg, 70%). **^{1}H NMR** (601 MHz, $CDCl_3$) δ/ppm = 7.35 (m, 5H, C_6**H_5**), 3.86 (s, 2H, C_6H_5C**H_2**S), 3.10 (s, 2H, SC**H_2**CO). **^{13}C NMR** (151 MHz, $CDCl_3$) δ/ppm = 176.40 (CH_2**C**OOH), 136.98 (**C_6**H_5), 129.36 (**C_6**H_5), 128.77 (**C_6**H_5), 127.58 (**C_6**H_5), 36.47 (S**C**H_2CO), 32.00 (C_6H_5**C**H_2S). **LC-MS-**

ES⁺(CH_3OH) found: 180.01. **HR-MS-ESI** (H_2O, ES-) cal.for $C_9H_9O_2S^-$ [M-H]$^-$: 181.0329; found: 181.0313.

S-benzyl-NHS-ester (2,5-Dioxo-1-pyrrolidinyl 2-(benzylthio)acetate, $C_{13}H_{13}NO_4S$ 279.31 g/mol)

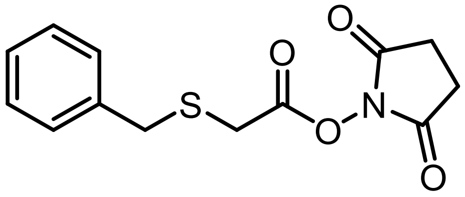

2-(benzylthio)acetic acid (12.7 mg, 0.071 mmol), *N*-hydroxysuccinimide (8.30 mg, 0.072 mmol) and DCC (14.9 mg, 0.072 mmol) was dissolved in DMF (0.40 mL). The reaction was stirred for 4 h. Then the reaction was filtered to remove solid and the solvent was removed under vacuum. The solid was further washed with EtOAc, finally yield the product as a white solid (17.8 mg, 91%). **^{1}H NMR** (601 MHz, $CDCl_3$) δ/ppm = 7.40 – 7.29 (m, 5H, $C_6\mathbf{H}_5$), 3.91 (s, 2H, $C_6H_5C\mathbf{H}_2S$), 3.29 (s, 2H, $SC\mathbf{H}_2CO$), 2.88 (s, 4H, $C\mathbf{H}_2C\mathbf{H}_2$). **^{13}C NMR** (151 MHz, $CDCl_3$) δ/ppm = 168.84 (O**C**-NO-**C**O), 165.79 (O**C**O), 136.37(**C**$_6H_5$), 129.33 (**C**$_6H_5$), 128.71 (**C**$_6H_5$), 127.54 (**C**$_6H_5$), 35.95 (S**C**H_2CO), 28.94 (C_6H_5**C**H_2S), 25.64 (**C**H_2**C**H_2). **LC-MS-ES⁺**(CH_3OH) found: 279.01; analytical data matches literature.[2]

benzyl 2-mercaptoacetate ($C_9H_{10}O_2S$, 182.04 g/mol)

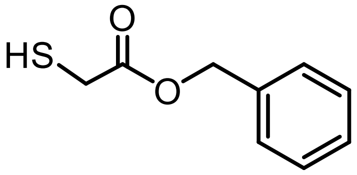

p-Toluenesulfonic acid (3.1 g, 18 mmol) was added dropwise to the DCM solution (250 mL) of BnOH (6.18 m, 60mmol) and 2-mercaptoacetic acid (5.1 mL 24 mmol). The reaction mixture was stirred at r.t. for 5 h. The mixture was washed with saturated sodium bicarbonate solution (450 mL), brine (600 mL), dried over Na_2SO_4, concentrated and purified by flash chromatography (petroleum ether: ethyl acetate .20:1) to give a colorless oil (6.1 g,.61%). **^{1}H NMR** (400 MHz, $CDCl_3$) δ 7.37 (s, 5H), 5.18 (s, 2H), 3.31 (d, *J* = 8.3 Hz, 2H), 2.02 (t, *J* = 8.3 Hz, 1H). **HR-MS-ESI** (CH_3OH, ES^-) cal.for $C_9H_9O_2S^-$ $[M-H]^-$: 181.0329; found: 181.0309.

Phenylmethyl 2-[(β-D-glucopyranosyl)thio]acetate ($C_{15}H_{20}O_7S$, 344.38 g/mol)

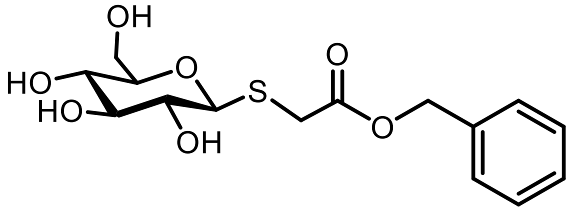

β-D-glucopyranose (50 mg, 0.28 mmol) was dissolved in water (2 mL). Triethylamine (0.84 mL, 6 mmol) was added, the reaction was cooled to 0 °C, and stirred. 2-Chloro-1,3-dimethylimidazolinium chloride (0.303 g, 1.8 mmol) was dissolved in benzyl 2-mercaptoacetate (0.125 g, 0.69 mmol) and the resulting solution was added dropwise to the aqueous β-D-glucopyranose solution over 2 min. The reaction was stirred at 0 °C for 0.5 h, and was then diluted with water (2 mL) and washed with CH_3Cl (4 x 10 mL). The aqueous layer was lyophilised, pre-adsorbed onto silica, and purified by flash column chromatography (CHCl:MeOH, 5:1) to afford a yellow oil (63 mg, 64%). **^{1}H NMR** (400 MHz, D_2O) δ/ppm = 7.44 (m, 5H), 5.22 (d, *J* = 5.0 Hz, 2H), **4.52 (d, *J* = 9.7 Hz, 1H, anomeric H)**,

3.78 (dd, J = 12.2, 1.9 Hz, 1H), 3.70 – 3.60 (m, 2H), 3.52 – 3.38 (m, 5H). **^{13}C NMR** (101 MHz, D_2O) δ 172.54, 135.28, 128.84, 128.75, 128.38, 84.50, 79.93, 77.12, 72.13, 69.33, 67.81, 60.76, 44.99, 31.32. **HR-MS-ESI** (CH_3OH, ES^+) cal.for $C_{15}H_{21}O_7S^+$ $[M+H]^+$: 345.1003; found: 345.1018; deviation: 4.49 ppm.

β-D-Glucosidothioglycolic acid ($C_8H_{14}O_7S$, 254.25 g/mol)

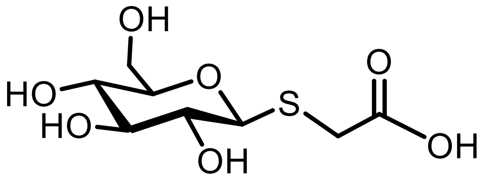

To a solution of phenylmethyl 2-[(β-D-glucopyranosyl)thio]acetate (100 mg, 0.29 mmol) in water (2 mL) was added NaOH (10 μL, 1 M) and left to stir for 2 h at rt, after which point the reaction was lyophilised and crude product was purified via column chromatography (EtOAc:IPA:H2O- 2:2:1) to yield the product as a white solid (37.7 mg, 51.1%); **^{1}H NMR** (400 MHz, D_2O) δ **4.57 (d, J = 9.8 Hz, 1H, anomeric H)**, 3.90 (d, J = 12.4 Hz, 1H), 3.72 (dd, J = 12.4, 4.9 Hz, 1H), 3.57 – 3.31 (m, 6H). **^{13}C NMR** (101 MHz, D_2O) δ 176.79, 84.76, 79.90, 77.06, 72.08, 69.43, 60.84, 33.28. **HR-MS-ESI** (H_2O, ES^+) cal.for $C_8H_{15}O_7S^+$ $[M+H]^+$: 255.0533; found: 255.0541.

Glc-NHS (2,5-Dioxo-1-pyrrolidinyl 2-[(β-D-glucopyranosyl)thio]acetate, $C_{12}H_{17}NO_9S$, 351.33 g/mol)

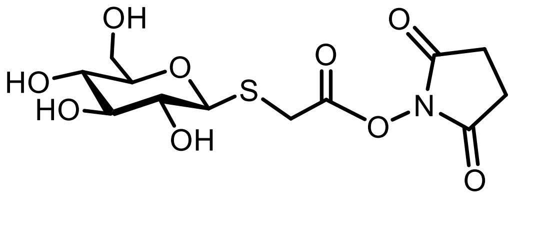

To a solution of Glc-SCH_2COOH (10.4 mg, 0.041 mmol) and *N*-hydroxysuccinimide (4.71 mg, 0.041 mmol) in DMF (350 μL) was added DCC (8.44 mg, 0.041 mmol). The reaction was vortexed and shaken at 40 °C, 400 rpm and the reaction was shaken for 4 h. After this

time, DMF was removed under vacuum and the residue resuspend in water, filtered with a 0.2 μm syringe filter, and lyophilised and finally purified by size exclusion and lyophilised once more and stored at -20°C and used as required, yielding the product as a white solid (13.9 mg, 96.7%); **^{1}H NMR** (400 MHz, D_2O) δ **5.09 (d, *J* = 9.9 Hz, 1H, anomeric H)**, 4.31 (t, *J* = 9.5 Hz, 1H,), 4.17 (d, *J* = 14.8 Hz, 1H), 3.96 – 3.86 (m, 2H), 3.75 (dd, *J* = 12.5, 5.0 Hz, 1H), 3.66 – 3.52 (m, 2H), 3.27 (d, *J* = 14.8 Hz, 1H), 2.80 (s, 4H). **^{13}C NMR** (101 MHz, D_2O) δ 176.51, 171.86, 81.02, 78.77, 76.75, 73.75, 69.60, 60.58, 26.02, 25.35, 25.17. **HR-MS-ESI** (CH_3OH, ES^-) cal.for $C_{12}H_{17}NO_9S^-$ $[M-H]^-$: 350.0551; found: 350.0533.

Acetobromo lactose (4-*O*-(2,3,4,6-tetra-*O*-acetyl-β-D-(+)-galactopyranosyl)-2,3,6-tri-*O*-acetyl-α-D-(+)-glucopyranosyl bromide, $C_{26}H_{35}BrO_{17}$, 699.45 g/mol**)**

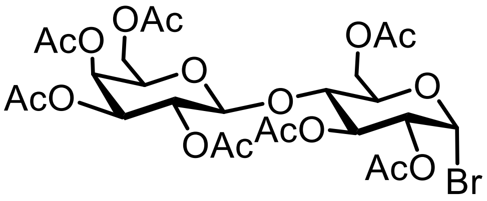

To a solution of lactose octaacetate (20 g, 29.4 mmol) in Ac_2O (8.35 mL, 88.4 mmol) at 0 °C was added HBr (25.8 mL, 147 mmol) dropwise and the reaction stirred for 40 min at 0 °C. After this time, the reaction was allowed to stir at room temperature for further 2 h. The reaction mixture was poured onto ice water (200 mL) and diluted with DCM (200 mL). The organic layer was separated and the aqueous phase extracted with DCM (100 mL). The combined aqueous extracts were washed with saturated $NaHCO_3$ (200 mL), 10 wt% $Na_2S_2O_3$ (200 mL) and brine (200 mL). The organic layer was then dried over anhydrous $MgSO_4$ and concentrated in vacuo to yield the product as a pale yellow syrup; 20.0 g, which was carried forward to the next step.

Ac-Lac-SAc (Thioacetyl 4-*O*-(2,3,4,6-tetra-*O*-acetyl-β-D-(+)-galactopyranosyl)-2,3,6-tri-*O*-acetyl-β-D-(+)-glucopyranoside, $C_{28}H_{38}O_{18}S$, 694.65 g/mol)

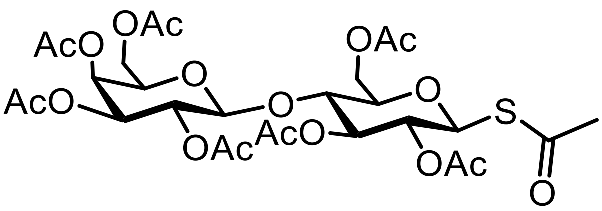

To a solution of 4-*O*-(2,3,4,6-tetra-*O*-acetyl-β-D-(+)-galactopyranosyl)-2,3,6-tri-*O*-acetyl-α-D-(+)-glucopyranosyl bromide (20 g, 28.6 mmol) in acetonitrile (100 mL) was added AcSK (6.53 g, 57.1 mmol) and left to stir overnight. The reaction mixture was diluted with DCM (100 mL) and washed with $NaHCO_3$ (2x200 mL), 1 M HCl (200 mL), water (2x200 mL) and brine (400 mL). The organic layer was dried over anhydrous $MgSO_4$ and concentrated in vacuo. The crude residue was purified via column chromatography (MeOH-CH_2Cl_2 0%→5% and EtOAc-petroleum ether 5%→90%) to yield the product as an off white solid (19.7 g, 90.0%). **^{1}H NMR** (400 MHz, $CDCl_3$) δ 5.34 (1H, dd, *J* = 3.5, 1.2 Hz, H-4b), 5.25 (1H, t, *J* = 9.0 Hz, H-3a), 5.20 (1H, d, *J* = 10.5 Hz, H-1a), 5.10 (1H, dd, *J* = 10.4, 7.9 Hz, H-2b), 5.03 (1H, dd, *J* = 10.4, 9.2 Hz, H-2a), 4.93 (1H, dd, *J* = 10.4, 3.5 Hz, H-3b), 4.45 (1H, d, *J* = 7.9 Hz, H-1b), 4.48 – 4.41 (1H, m, H-6a), 4.16 – 4.03 (3H, m, 2x H-6b, H-6a), 3.86 (1H, td, *J* = 6.9, 1.2 Hz, H-5b), 3.83 – 3.78 (1H, m, H-4a), 3.74 (1H, ddd, *J* = 10.0, 4.7, 1.9 Hz, H-5a), 2.36 (3H, s, CH_3), 2.14 (3H, s, CH_3), 2.10 (3H, s, CH_3), 2.06 (3H, s, CH_3), 2.04 (3H, s, CH_3), 2.03 (3H, s, v), 2.01 (3H, s, CH_3), 1.95 (3H, s, CH_3); **^{13}C NMR** (101 MHz, $CDCl_3$) δ 191.89 (CO), 170.38 (CO), 170.34 (CO), 170.16 (CO), 170.08 (CO), 169.63 (CO), 169.60 (CO), 169.00 (CO), 100.91 (C-1b), 80.02 (C-1a), 77.16 (C-5a), 75.74 (C-4a), 73.67 (C-4b), 70.99 (C-3b), 70.73 (C-5b), 69.27 (C-2a), 68.96 (C-2b), 66.62 (C-4b), 61.97 (C-6a), 60.90 (C-6b), 30.86 (CH_3), 20.88 (CH_3), 20.77 (CH_3), 20.67 (CH_3), 20.65 (CH_3), 20.64 (CH_3), 20.53 (2xCH_3). **LRMS:** m/z (ES+) 717.2 (100%, $[M+Na]^+$).

Ac-Lac-SCH2COOH ($C_{28}H_{38}O_{19}S$, 710.65 g/mol)

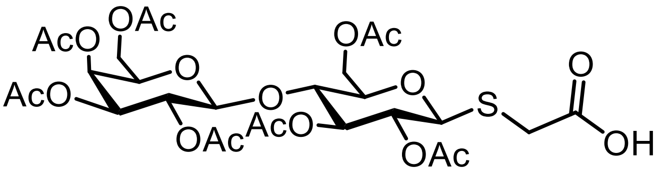

To a solution of thioacetyl 4-*O*-(2,3,4,6-tetra-*O*-acetyl-β-D-(+)-galactopyranosyl)-2,3,6-tri-*O*-acetyl-β-D-(+)-glucopyranoside (1.00 g, 1.44 mmol) in DMF (15 mL) was added hydrazine acetate (159 mg, 1.73 mmol), bromoacetic acid (300 mg, 2.16 mmol) and NEt_3 (0.6 mL, 4.32 mmol) and the reaction was left to stir for 1 h. After this time, the solvent was removed under high vacuum and the residue dissolved in DCM (50 mL) and washed with saturated $NaHCO_3$ (30 mL). The aqueous was extracted with DCM (2x 20 mL) and the combine organics washed with 1 M HCl (50 mL) and brine (50 mL). The organic layer was dried over anhydrous $MgSO_4$ and concentrated in vacuo. The crude residue was purified via column chromatography (MeOH-$CHCl_3$ 0%→20%) to yield the product as white solid (794 mg, 77.7%). **^{1}H NMR** (400 MHz, $CDCl_3$) δ 5.34 (1H, dd, *J* = 3.5, 1.2 Hz, H-4b), 5.22 (1H, t, *J* = 9.2 Hz, H-3a), 5.09 (1H, dd, *J* = 10.4, 7.9 Hz, H-2b), 4.97 (1H, dd, *J* = 10.2, 9.2 Hz, H-2a), 4.95 (1H, ds, *J* = 10.4, 3.5 Hz H-3b), 4.64 (1H, d, *J* = 10.1 Hz, H-1a), 4.55 (1H, dd, *J* = 12.1, 2.0 Hz, H-6a), 4.49 (1H, d, *J* = 7.9 Hz, H-1b), 4.15 – 4.03 (3H, m, 2x H-6b, H-6a) 3.88 1H, td, *J* = 6.8, 1.2 Hz, H-5b), 3.80 (1H, t, *J* = 9.5 Hz, H-4a), 3.62 (1H, ddd, *J* = 10.0, 4.9, 2.0 Hz, H-5a), 3.49 (1H, d, *J* = 15.2 Hz, SCH_2), 3.26 (1H, d, *J* = 15.2 Hz, SCH_2), 2.14 (3H, s, CH_3), 2.13 (3H, s, CH_3), 2.06 (3H, s, CH_3), 2.05 (3H, s, CH_3), 2.04 (3H, s, CH_3), 2.03 (3H, s CH_3), 1.96 (3H, s, CH_3); **^{13}C NMR** (101 MHz, $CDCl_3$) δ 170.41 (CO), 170.18 (CO), 170.11 (CO), 169.78 (CO), 169.71 (CO), 169.05 (CO), 162.98 (CO), 101.01 (C-1b), 82.22 (C-1a), 76.96 (C-5a), 75.89 (C-4a), 73.49 (C-3a), 70.95 (C-3b), 70.71 (C-5b), 69.89 (C-2a), 69.08 (C-2b), 66.63 (C-4b), 61.72 (C-6b) 60.82 (C-6a), 20.97 (CH_3), 20.80 (CH_3), 20.69 (CH_3), 20.67 (CH_3), 20.66 (CH_3), 20.53 (CH_3), 20.53 (CH_3). **LRMS** (ES$^+$): 733.2 (100%, $[M+Na]^+$).

Lac-SCH_2COOH ($C_{14}H_{24}O_{12}S$, 416.39 g/mol)

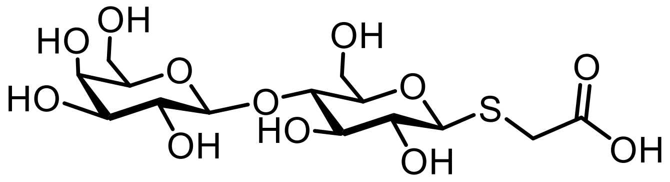

To a solution of Ac-Lac-SCH2COOH (500 mg, 0.704 mmol) in MeOH (15 mL) was added NaOMe (0.14 mL, 30 wt%, 0.774 mmol) and the reaction was stirred for 1 h. After this time, the reaction was neutralised with Dowex 50WX8 100-200 mesh exchange resin, filtered and the solvent removed under vacuum to yield the product as a white solid (280 mg, quantitative yield). **^{1}H NMR** (400 MHz, D_2O) δ 4.58 (1H, d, *J* = 9.9 Hz, H-1a), 4.43 (1H, d, *J* = 7.8 Hz, H-1b), 3.93 (1H, dd, *J* = 12.3, 2.2 Hz, H-6a), 3.90 (1H, d, *J* = 3.4 Hz, H-4a), 3.77 (1H, dd, *J* = 12.4, 4.8 Hz, H-6a), 3.75 (2H, d, *J* = 11.2 Hz, H-6b), 3.71 – 3.67 (2H, m, H-4b, H-5b), 3.64 (1H, dd, *J* = 10.1, 3.7 Hz, H-3a), 3.62 (2H, dd, *J* = 9.2, 8.4 Hz, H-3b), 3.58 – 3.54 (m, 1H, H-5a), 3.52 (1H, d, *J* = 15.3 Hz, SCH_2), 3.51 (1H, dd, *J* = 10.0, 7.8 Hz, H-2b), 3.41 (1H, d, *J* = 15.3 Hz, SCH_2), 3.39 (1H, dd, *J* = 9.9, 8.7 Hz, H-2a); **^{13}C NMR** (101 MHz, D_2O) δ 176.05 (CO), 102.87 (C-1b), 84.69 (C-1a), 78.74 (C-5a), 77.90 (C-4b/5b), 75.65 (C-3a/3b), 75.37 (C-4b/5b), 72.52 (C-3a/b), 71.84 (C-2a), 70.97 (C-2b), 68.58 (C-4a), 61.06 (C-6b), 60.08 (C-6a), 32.67 (SCH_2). **LRMS:** m/z (ES+) 439.0 (100%, $[M+Na]^+$).

Gal-Glc-NHS (α-lactosylthio NHS acetate, $C_{18}H_{27}NO_{14}S$, 513.47 g/mol)

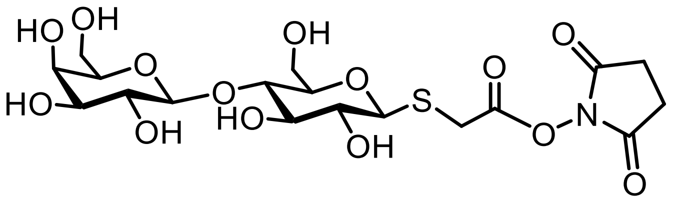

To a solution of Lac-SCH_2COOH (300 mg, 0.721 mmol) and *N*-hydroxysuccinimide (83.0 mg, 0.721 mmol) in DMF (2 mL) was added DCC (149 mg, 0.721 mmol) dissolved in DMF (2 mL) and the reaction was stirred for 4 h. After this time, the reaction filtered to remove solid and the solvent removed under vacuum and the solid further washed with EtOAc and petrol to yield the product as a white solid (351 mg, quantitative yield); **^{1}H NMR** (400 MHz, D_2O) δ 5.12 (1H, d, *J* = 9.9 Hz, H-1a), 4.48 (1H, d, *J* = 7.7 Hz, H-1b), 4.37 (1H, t, *J* = 9.6 Hz, H-2a), 4.18 (1H, d, *J* = 14.9 Hz, SCH_2), 4.06 (1H, t, *J* = 8.9 Hz, H-3a), 3.98 (1H, dd, *J* = 12.5, 2.2 Hz, H-6), 3.94 (1H, d, *J* = 3.4 Hz, H-4b), 3.88 – 3.73 (6H, m, 2x H-6, H-6, H-5a, H-5b, H-5a), 3.68 (1H, dd, *J* = 10.0, 3.5 Hz, H-3b), 3.57 (1H, dd, *J* = 11.7, 6.0 Hz, H-2b), 3.28

(1H, d, J = 14.8 Hz, SCH_2), 2.95 (2H, a d J = 11.0 Hz, 2H, $NCOCH_2$), 2.80 (s, 2H, $NCOCH_2$). **^{13}C NMR** (101 MHz, D_2O) δ 179.03 (CO), 174.08 (CO), 105.50 (C-1b), 82.28 (C-5a/b), 80.90(C-5a/b), 80.81 (C-2a), 79.14 (C-1a), 77.90 (C-3a), 75.04 (C-3a), 75.00 (C-3b), 73.39 (C-2b), 71.02 (C-4b), 63.48 (C-6a/b), 62.47 (C-6a/b), 28.51 (SCH), 27.87 (NCO*C*H_2), 27.69 (NCO*C*H_2). **LRMS** (ES^-): found 512.0 (100%, $[M-H]^-$).

Sia-Gal-Glc-SCH_2COOH ($C_{25}H_{41}NO_{20}S$, 707.65 g/mol)

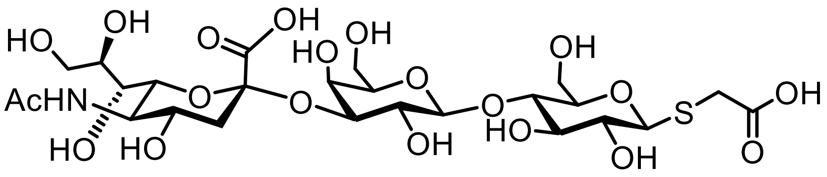

To a solution of Lac-SCH_2COOH (200 mg, 0.48 mmol) in Buffer (48 mL, 100 mM $(NH_4)_2CO_3$, 20 mM $MgCl_2$, pH 8.5) was added CTP disodium salt (632 mg, 1.2 mmol), Neu5Ac (222 mg, 0.72 mmol), *Pasteurella multocida* Sialyltransferase (2.5 mg, 710 μL, 3.52 mg/mL) and *Neisseria meningitides* CMP-sialic acid synthetase (3 mg, 1.075 μL, 2.79 mg/mL) and reaction shaken at 37 °C 225 rpm. After 3 h, TLC indicated no further reaction. The solution was concentrated and lyophilised to give the crude solid which was subjected to LH-20 size exclusion and fraction with the desired compound were lyophilised to give a crude white solid. **^{1}H NMR** (601 MHz, D_2O) δ 4.55 (1H d, J = 9.9 Hz), 4.52 (1H, d, J = 7.9 Hz), 4.11 (1H, dd, J = 9.9, 3.3 Hz), 3.96 (2H, dt, J = 6.6, 2.8 Hz), 3.91 – 3.53 (12H, m), 3.46 – 3.33 (3H, m), 2.75 (1H, dd, J = 12.3, 4.6 Hz), 2.02 (3H, s), 1.80 (1H, t, J = 12.2 Hz). **^{13}C NMR** (101 MHz, D_2O) δ 177.27, 175.06, 173.91, 145.67, 102.65, 99.86, 84.72, 78.78, 78.01, 75.62, 75.52, 75.18, 72.92, 71.88, 71.80, 69.41, 68.39, 68.17, 67.54, 62.63, 61.06, 60.21, 51.75, 39.68, 33.84, 22.09. **HR-MS-ESI** (CH_3OH, ES^-) cal.for $C_{25}H_{40}NO_{20}S^-$ $[M-H]^-$: 706.1870; found: 706.1844.

General procedure for activated ester formation of Sia-Gal-Glc-SCH_2COOH

Sia-Gal-Glc-SCH_2COOH was dissolved in a solution of NHS, NHSS or PFP (10 mg/mL in DMF) at the desired equivalents and vortexed followed by DCC or EDC (10 mg/mL in

DMF), vortexed again and incubated at the desired temperature at 500 rpm or more. After overnight reaction the DMF was removed under vacuum and the residue resuspend in water, filtered with a 0.2 μm syringe filter, and lyophilised and finally purified by G10, G25 or LH20 resin and used as required for reactions with HEL.

Sia-Gal-Glc-NHS (α-Neu5Ac-(2→3)-α-lactosylthio NHS acetate, $C_{29}H_{44}N_2O_{22}S$, 804.72 g/mol)

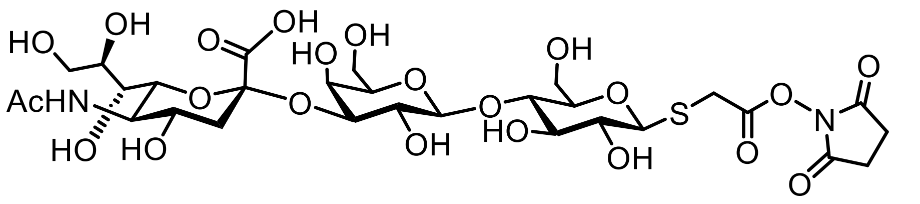

Sia-Gal-Glc-SCH_2COOH (2 mg, 2.83 nmol) was dissolved in a solution of NHS (65 μL, 10 mg/mL in DMF, 2 eq) and vortexed followed by the addition of EDC (54.2 μL, 10 mg/mL in DMF, 1 eq). The reaction was vortexed and the volume was bought to 200 μL with DMF and finally water (10 μL) was added, vortexed again and incubated at 25 °C for at 500 rpm 1 h after which time the temperature was increased to 40 °C for 1 h. After this time, DMF was removed under vacuum and the residue resuspend in water, filtered with a 0.2 μm syringe filter, and lyophilised and finally purified by size exclusion and lyophilised once more and stored at -20°C and used as required. ^1H NMR conversion (D_2O): 29.6%.

Sia-Gal-Glc-NHSS (α-Neu5Ac-(2→3)-α-lactosylthio N-hydroxysulfosuccinimide ester, $C_{29}H_{43}N_2NaO_{25}S_2$, 906.76 g/mol)

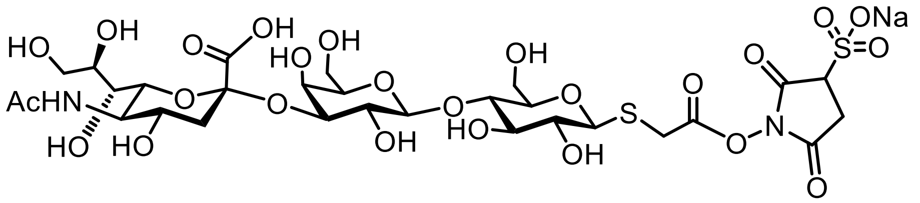

Sia-Gal-Glc-SCH_2COOH (2 mg, 2.83 nmol) was dissolved in a solution of NHSS (122.7 μL, 10 mg/mL in 10:1 DMF:H_20, 2 eq) and vortexed followed by addition of EDC (54.2 μL, 10 mg/mL in DMF, 1 eq). The reaction was vortexed and the volume was bought to 200 μL

with DMF and finally water (10 μL) was added, vortexed again and incubated at 25 °C for at 500 rpm 1 h after which time the temperature was increased to 40 °C for 1 h. After this time, DMF was removed under vacuum and the residue resuspend in water, filtered with a 0.2 μm syringe filter, and lyophilised and finally purified by size exclusion and lyophilised once more and stored at -20°C and used as required. ^{1}H NMR conversion (D_2O): 39.0%

Sia-Gal-Glc-S-PFP (α-Neu5Ac-(2→3)-α-lactosylthio pentafluorophenyl ester, $C_{31}H_{40}F_5NO_{20}S$, 873.70 g/mol)

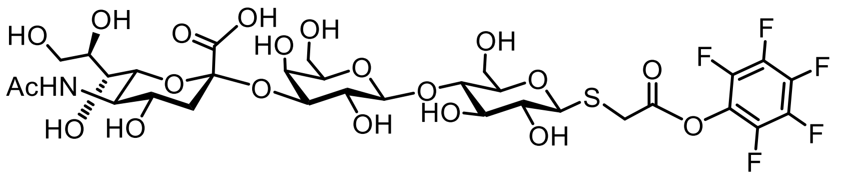

Sia-Gal-Glc-SCH$_2$COOH (2 mg, 2.83 nmol) was dissolved in a solution of NHS (105.9 μL, 10 mg/mL in DMF, 2 eq) and vortexed and followed by EDC (54.2 μL, 10 mg/mL in DMF, 1 eq). The reaction was vortexed and the final volume was bought to 200 μL with DMF and finally water (10 μL) was added, vortexed again and incubated at 25 °C for at 500 rpm 1 h after which time the temperature was increased to 40 °C for 1 h. After this time, DMF was removed under vacuum and the residue resuspend in water, filtered with a 0.2 μm syringe filter, and lyophilised and finally purified by size exclusion and lyophilised once more and stored at -20°C and used as required. ^{1}H NMR conversion (D_2O): 12.3%

2-(Acetylthio)acetic acid ($C_4H_6O_3S$, 134.15 g/mol)

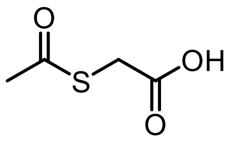

Sodium bicarbonate (3.14g, 37.4 mmol) was added snowly to a stirred solution of thioacetic acid (1.62g, 21.4 mmol) and Bromoacetic acid (2.24g, 16.0 mmol) in 20 mL water, stirred at r.t. overnight. The resulting reaction solution was acified to pH=1.0 with concentrated HCl,

extracted with EtOAc (3x8mL). Water (3x20 mL) was added to wash the EtOAc phase, then dried with $MgSO_4$, filtered and evaporated to give yellow oil.^{1}**H NMR** (601 MHz, $CDCl_3$) δ/ppm = 3.74 (s, 2H), 2.41 (s, 3H). ^{13}**C NMR** (151 MHz, $CDCl_3$) δ/ppm = 194.01, 174.41, 31.36, 30.24. **LRMS** (ES^+) *m/z*: 178.9 (100%, M+2Na^+). Analytical data matches literature.[3]

SATA-NHSS (1-(2-(acetylthio)acetoxy)-2,5-dioxopyrrolidine-3-sulfonic acid, $C_8H_9NO_8S_2$, 311.28 g/mol)

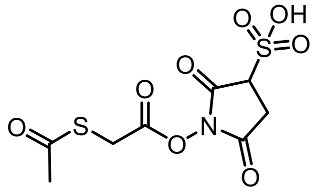

A solution of 0.88 M dicyclohexylcarbodiimide in DMF (41.3 mg, 0.2 mmol, in 250 µL DMF) was added to a suspension of 43.4 mg sodium *N*-hydroxysulfosuccinimide (200 µmol) and 26.8 mg 2-(Acetylthio)acetic acid (200 µmol) in 250 µL DMF in a 2.0 mL microcentrifuge tube. The mixture was incubated 16.5 hr, 800 rpm at RT, during which time the sodium *N*-hydroxysulfosuccinimide dissolved and another precipitate formed. After cooling at 4 °C for 2.5 h, the suspension was centrifuged (20,000 x g, 10 min) and the supernatant transferred to a 50 mL centrifuge tube. The pellet was washed twice with 400 µL DMF. The washes were pooled with the original supernatant to give a total volume of 1.3 mL. Ethyl acetate (30 mL) was added, and a precipitate was allowed to form over 1 hour. The precipitate was collected by centrifugation (4000 x g, 10 min). The pellet was washed successively with 20 mL of ethyl acetate (2x) and 15 mL of ethyl ether (3x), then dried. ^{1}**H NMR** (400 MHz, DMSO-d6) δ/ppm 4.18 (s, 2H, S-C**H**$_2$-CO), 3.71 (dd, *J* = 8.7, 2.4 Hz, 1H, -C**H**-), 2.87 (d, *J* = 8.7 Hz, 1H, -C**H**$_2$-), 2.68 (dd, *J* = 18.0, 2.4 Hz, 1H, -C**H**$_2$-), 2.44 (s, 3H, C**H**$_3$-CO). For peak assignment, the starting material of sodium *N*-hydroxysulfosuccinimide was also identified by ^{1}H NMR (400 MHz, DMSO-d6): δ 3.70 (ddt, *J* = 7.6, 5.3, 2.3 Hz, 1H), 2.95 – 2.83 (m, 1H), 2.67 (dt, *J* = 17.9, 2.1 Hz, 1H). ^{13}**C NMR** (101 MHz, DMSO-d6) δ/ppm = 193.72,

168.90, 168.53, 165.42, 56.66, 31.18, 30.38, 28.58. **HR-MS-ESI** (CH_3OH, ES^-) cal.for $C_8H_8NO_8S_2^-$ $[M-H]^-$: 309.9697; found: 309.9695.

Supplementary References

1. Ning, X. et al. Design, synthesis, and biological evaluation of (e)-3,4-dihydroxystyryl aralkyl sulfones and sulfoxides as novel multifunctional neuroprotective agents. *J Med Chem* **57**, 4302-4312 (2014).

2. Dengler, S., Douat, C. & Huc, I. Differential Peptide Multi-Macrocyclizations at the Surface of a Helical Foldamer Template. *Angew Chem Int Ed Engl* **61**, e202211138 (2022).

3. Lv, P. et al. Design, Synthesis, and Antifungal Activities of 3-Acyl Thiotetronic Acid Derivatives: New Fatty Acid Synthase Inhibitors. *J Agric Food Chem* **66**, 1023-1032 (2018).
