# Supplementary material for: Thio-NHS esters are non-innocent protein acylating reagents
Source: Nat Commun. 2025 Jul 1;16:6028. doi: 10.1038/s41467-025-60527-5 (PMC12217259; doi:10.1038/s41467-025-60527-5)
Supplement: Supplementary file 2 — Reporting Summary [file 41467_2025_60527_MOESM2_ESM.pdf]

nature portfolio

Corresponding author(s): Mohammed, Davis

Last updated by author(s): May 24, 2025

Reporting Summary

Nature Portfolio wishes to improve the reproducibility of the work that we publish. This form provides structure for consistency and transparency in reporting. For further information on Nature Portfolio policies, see our Editorial Policies and the Editorial Policy Checklist.

Statistics

For all statistical analyses, confirm that the following items are present in the figure legend, table legend, main text, or Methods section.

n/a	Confirmed	
☐	☒	The exact sample size (*n*) for each experimental group/condition, given as a discrete number and unit of measurement
☐	☒	A statement on whether measurements were taken from distinct samples or whether the same sample was measured repeatedly
☐	☒	The statistical test(s) used AND whether they are one- or two-sided<br>*Only common tests should be described solely by name; describe more complex techniques in the Methods section.*
☒	☐	A description of all covariates tested
☐	☒	A description of any assumptions or corrections, such as tests of normality and adjustment for multiple comparisons
☐	☒	A full description of the statistical parameters including central tendency (e.g. means) or other basic estimates (e.g. regression coefficient) AND variation (e.g. standard deviation) or associated estimates of uncertainty (e.g. confidence intervals)
☐	☒	For null hypothesis testing, the test statistic (e.g. *F*, *t*, *r*) with confidence intervals, effect sizes, degrees of freedom and *P* value noted<br>*Give P values as exact values whenever suitable.*
☒	☐	For Bayesian analysis, information on the choice of priors and Markov chain Monte Carlo settings
☒	☐	For hierarchical and complex designs, identification of the appropriate level for tests and full reporting of outcomes
☒	☐	Estimates of effect sizes (e.g. Cohen's *d*, Pearson's *r*), indicating how they were calculated

Our web collection on statistics for biologists contains articles on many of the points above.

Software and code

Policy information about availability of computer code

Data collection	Data was collected using the software provided by the respective instrument vendor and is specified in the Methods section.
Data analysis	Data was analysed with MassLynx 4.2 (Waters), GraphPad Prism 10 (GraphPad Software Inc.), MestReNova-14.1.1-24571 (Mestrelab Research S.L.), Microsoft Excel 16 (Microsoft, version 16.0.17830.20056), MaxQuant (v.2.3.0.0), MSFragger-based FragPipe (FragPipe interface (v21.1) was used with MSFragger (version: 4.0), Philosopher (version: 5.1.0), IonQuant (version 1.10.12)4 and Python (version: 3.9.18) enabled), AlphaFold 2(open source, version 2.0). Protein subcellular localzation analysis was enabled by Gene Ontology (G0) analysis from DAVID Biointormatics Resources. Data processing were performed using Image Lab (Bio-Rad Laboratories v6.1), ImageJ (1.54d), Python (3.10.11) and R (open source, 4.4.0).

For manuscripts utilizing custom algorithms or software that are central to the research but not yet described in published literature, software must be made available to editors and reviewers. We strongly encourage code deposition in a community repository (e.g. GitHub). See the Nature Portfolio guidelines for submitting code & software for further information.

Data

Policy information about availability of data

All manuscripts must include a data availability statement. This statement should provide the following information, where applicable:

- Accession codes, unique identifiers, or web links for publicly available datasets
- A description of any restrictions on data availability
- For clinical datasets or third party data, please ensure that the statement adheres to our policy

The mass spectrometry data for proteomics and modification site identification generated in this study have been deposited in the ProteomeXchange Consortium through the PRIDE77 partner repository, with the dataset identifier PXD056955 [http://proteomecentral.proteomexchange.org/cgi/GetDataset?ID=PXD056955] and PXD056949 [http://proteomecentral.proteomexchange.org/cgi/GetDataset?ID=PXD056949]. Source data are provided with this paper. Any further data supporting the findings of this study are available from the corresponding author upon request

Research involving human participants, their data, or biological material

Policy information about studies with human participants or human data. See also policy information about sex, gender (identity/presentation), and sexual orientation and race, ethnicity and racism.

Reporting on sex and gender	N/A
Reporting on race, ethnicity, or other socially relevant groupings	N/A
Population characteristics	N/A
Recruitment	N/A
Ethics oversight	N/A

Note that full information on the approval of the study protocol must also be provided in the manuscript.

Field-specific reporting

Please select the one below that is the best fit for your research. If you are not sure, read the appropriate sections before making your selection.

[x] Life sciences [ ] Behavioural & social sciences [ ] Ecological, evolutionary & environmental sciences

For a reference copy of the document with all sections, see nature.com/documents/nr-reporting-summary-flat.pdf

Life sciences study design

All studies must disclose on these points even when the disclosure is negative.

Sample size	Validation of NHS esters modification and LC-MS analysis was conducted carried out independently with n=3. Synthetic reactions and protein modifications were carried out independently for multiple times and proved to be reliable and consistent. The reported values for conversion and yield should be regarded as semi-quantitative.<br>For proteomic analysis, the sample size was determined by the number of confidently identified lysine modification sites detected via LC-MS/MS. No statistical methods were used to predetermine sample size. Sites with low-confidence structural predictions (AlphaFold prediction quality ≤70) were excluded. The number of modification sites was sufficient for statistical comparisons.
Data exclusions	No data was excluded.
Replication	Protein modification NHS esters, LC-MS analysis, and Proteomic experiments were repeated at least twice with similar results and one representative result was shown in the study.
Randomization	During mass spectrometric measurements, liquid chromatography columns were routinely cleaned between runs, and replicate samples from the same experimental set were injected in a random order to prevent measurement biases. Additionally, batch effects and experimental biases were further minimized during downstream computational analyses through normalization and cross-validation of the dataset. For proteomic experiments, sample allocation is not applicable.
Blinding	For all experiments, including reaction of NHS esters and proteomic data were collected and analyzed without bias.

Reporting for specific materials, systems and methods

We require information from authors about some types of materials, experimental systems and methods used in many studies. Here, indicate whether each material, system or method listed is relevant to your study. If you are not sure if a list item applies to your research, read the appropriate section before selecting a response.

Materials & experimental systems

n/a	Involved in the study
☒	☐ Antibodies
☒	☐ Eukaryotic cell lines
☒	☐ Palaeontology and archaeology
☒	☐ Animals and other organisms
☒	☐ Clinical data
☒	☐ Dual use research of concern
☒	☐ Plants

Methods

n/a	Involved in the study
☒	☐ ChIP-seq
☒	☐ Flow cytometry
☒	☐ MRI-based neuroimaging

Plants

Seed stocks	N/A
Novel plant genotypes	N/A
Authentication	N/A
